# Proteomic Analysis of Disease Stratified Human Pancreas Tissue Indicates Unique Signature of Type 1 Diabetes

**DOI:** 10.1371/journal.pone.0135663

**Published:** 2015-08-24

**Authors:** Tanya C. Burch, Margaret A. Morris, Martha Campbell-Thompson, Alberto Pugliese, Jerry L. Nadler, Julius O. Nyalwidhe

**Affiliations:** 1 Department of Microbiology and Molecular Cell Biology, Eastern Virginia Medical School, Norfolk, VA, United States of America; 2 Leroy T. Canoles Jr. Cancer Research Center, Eastern Virginia Medical School, Norfolk, VA, United States of America; 3 Department of Internal Medicine, Eastern Virginia Medical School, Norfolk, VA, United States of America; 4 Strelitz Diabetes Research Center, Eastern Virginia Medical School, Norfolk, VA, United States of America; 5 Department of Pathology, Immunology, and Laboratory Medicine, College of Medicine, University of Florida Gainesville, FL, United States of America; 6 Diabetes Research Institute, Miller School of Medicine, University of Miami, Miami, FL, United States of America; La Jolla Institute for Allergy and Immunology, UNITED STATES

## Abstract

Type 1 diabetes (T1D) and type 2 diabetes (T2D) are associated with functional beta cell loss due to ongoing inflammation. Despite shared similarities, T1D is an autoimmune disease with evidence of autoantibody production, as well as a role for exocrine pancreas involvement. Our hypothesis is that differential protein expression occurs in disease stratified pancreas tissues and regulated proteins from endocrine and exocrine tissues are potential markers of disease and potential therapeutic targets. The study objective was to identify novel proteins that distinguish the pancreas from donors with T1D from the pancreas from patients with T2D, or autoantibody positive non-diabetic donors. Detailed quantitative comprehensive proteomic analysis was applied to snap frozen human pancreatic tissue lysates from organ donors without diabetes, with T1D-associated autoantibodies in the absence of diabetes, with T1D, or with T2D. These disease-stratified human pancreas tissues contain exocrine and endocrine tissues (with dysfunctional islets) in the same microenvironment. The expression profiles of several of the proteins were further verified by western blot. We identified protein panels that are significantly and uniquely upregulated in the three disease-stratified pancreas tissues compared to non-disease control tissues. These proteins are involved in inflammation, metabolic regulation, and autoimmunity, all of which are pathways linked to, and likely involved in, T1 and T2 diabetes pathogenesis. Several new proteins were differentially upregulated in prediabetic, T1D, and T2D pancreas. The results identify proteins that could serve as novel prognostic, diagnostic, and therapeutic tools to preserve functional islet mass in Type 1 Diabetes.

## Introduction

Type 1 diabetes (T1D) is a chronic, inflammatory disease widely considered to result from the autoimmune destruction of the insulin-producing pancreatic beta cells, leading to severe insulin deficiency and chronic hyperglycemia [1–3]. Presently, no therapy effectively prevents or reverses T1D, and patients must endure lifelong insulin replacement therapy with risk of severe complications. The progression of T1D is modulated by a complex interplay between beta cells, the immune system, and the environment in genetically susceptible individuals. Human leukocyte antigen genotypes and islet autoantibodies are currently the most useful biomarkers for T1D risk prediction. Serological appearance of one or more autoantibodies against islet cell antigens (i.e., glutamic acid decarboxylase, insulin, protein tyrosine phosphatase, and zinc transporter Slc30A8 protein) is among the first detectable signs of emerging beta cell autoimmunity [4–5]. Autoantibodies to these molecules are both diagnostic and prognostic of disease development; however, routine use of autoantibody levels as a diagnostic tool is cumbersome, at best. Prevention trials to stop or slow the natural progression of T1D could be designed and executed better if we had available robust biomarkers of the processes that ultimately are at the origin of the disease [6–7]. Due to the limitations of current biomarkers, there is an unmet clinical need to identify novel T1D biomarkers to improve the sensitivity and specificity of T1D prediction and disease monitoring after treatment.

Additionally, a critical challenge in diagnosing T1D is the increasing rate of type 2 diabetes (T2D) in young patients [8]. T2D represents a highly complex and heterogeneous disease that is influenced by both genetic and environmental factors. Insulin resistance is a primary defect in T2D, where the uptake of glucose into muscle is impaired. In addition loss of beta cell mass has been seen in T2D [9–11]. Proposed mechanisms to explain the insulin resistance and islet β-cell dysfunction and loss are oxidative stress, endoplasmic reticulum stress, amyloid deposition in the pancreas, ectopic lipid deposition in the muscle, liver and pancreas, and general lipotoxicity and glucotoxicity.

Mass spectrometry-based proteomics is a high-throughput and highly sensitive technique for analyzing complex biological samples. Appropriately designed and interpreted global, unbiased, top-down proteomic studies are well suited to the study of the pathogenic mechanisms of T1D, and the identification of biomarkers for the disease. The discovery of proteins that are specifically altered in T1D pancreas would identify novel disease associated pathways. Some these proteins may be novel diagnostic and prognostic disease biomarkers and therapeutic targets. The pancreas is a complex multifunctional gland, with exocrine and endocrine activities that are required to control nutrition balance. The endocrine pancreas islets of Langerhans contain alpha and beta cells, which synthesize and secrete the blood glucose level regulating hormones, glucagon and insulin, respectively. Furthermore, recent evidence has suggested a role of the exocrine pancreas in T1D [12]. To date, no detailed global comprehensive proteomics study of disease stratified human pancreas, containing exocrine and endocrine tissues (with dysfunctional islets) in the same microenvironment, has been performed. The emphasis has been on the analysis of isolated human islets cultured *in vitro*, laser capture microdissected islets, islets obtained from primary cultures and *in vivo* animal models [13–19]. The goal of this study was to use innovative mass spectrometry techniques to profile and identify potential exocrine and endocrine pancreas tissue-based biomarkers that distinguish T1D patients from normal control, AAb+ (non-diabetic, autoantibody positive patients), and T2D patients using human samples from the Network for Organ Donors with Diabetes (nPOD).

## Materials and Methods

### Study Approval and Human Pancreatic Tissue

The pancreatic specimens were from deceased organ donors provided by nPOD, University of Florida, Gainesville, Florida, USA, in accordance with ethical regulations [20–21]. These samples were considered not to involve human subjects, and deemed exempt by the Eastern Virginia Medical School Institutional Review Board. Specimens were from four cohorts: non-diabetic donors (ND), non-diabetic donors with T1D-associated autoantibodies (AAb+), donors with T1D and donors with T2D. The present work focused on the proteomic analysis of 5 cases from each group. The nPOD cases classification criteria are described under S1 Table.

### Experimental Design

Pancreas tissues were processed and digested with trypsin to generate peptides that were subjected to mass spectrometry on a Q-Exactive mass spectrometer. MS/MS spectra were searched against the SwissProt database for protein identification. Protein quantitation was determined by average total ion intensity using Scaffold Q+S (Proteome Software Inc., Portland, OR). The experimental work-flow is summarized in S1 Fig.

### Database Searching, Protein Identification and Quantitation

Tandem mass spectra were extracted by Proteome Discoverer 1.9 and the MS/MS spectra searched against the Sprot_2014_11 database using Mascot (Matrix Science, UK). The search parameters were: taxonomy, humans and viruses; enzyme, trypsin with a maximum of 1 missed cleavage site; parent and fragment ion mass tolerances, 20 ppm and 80mmu respectively; carbamidomethylated cysteines; methionine oxidation variable modification. Searches were also performed against the reversed concatenated SwissProt database and filtered to a < 1% false discovery rate (FDR). Scaffold was used to validate MS/MS based peptide and protein identifications. Peptide identifications with a probability score greater than 95.0% were accepted by Peptide Prophet [22], with Scaffold delta-mass correction. Protein identifications with a probability score greater than 99.0% and if they contained at least 2 identified peptides using the Protein Prophet algorithm [23]. Proteins that contained similar peptides and could not be differentiated based on MS/MS analysis alone were grouped to satisfy the principles of parsimony.

ScaffoldQ + S was used to perform average total ion chromatogram label free quantitation to determine differential protein expression between the four different sample groups [24] The quantitation methods are described under S1 Fig and in S1 File. Proteins were considered to be differentially regulated between the sample cohorts if they complied with the following parameters: FDR <0.05 and fold change (FC) ≥ 2.0, with p- values < 0.05 based on the Scaffold algorithm [25]. Proteins were annotated with GO terms from NCBI (downloaded May 2, 2014) [26]. Functional annotation of these proteins was done using David [27] and Panther [28]. Interactions networks and pathway analysis was performed using Ingenuity Pathway Analysis (QIAGEN).

### Validation of Differentially Expressed Proteins

Protein concentration was measured by BCA assay. Normalized protein concentrations (40μg) from pooled sample cohorts were separated by SDS-PAGE and transferred to PDVF membranes and processed using standard methods. The corresponding full length recombinant proteins were included as positive controls in the analyses. The membranes were incubated with the respective primary antibodies at 4°C overnight. The source and dilutions of the antibodies and the recombinant proteins are summarized in S2 table. The membranes were washed extensively with 0.1% Tween-20 in PBS before incubation with species-specific IRDye700 or 800-conjugated secondary antibodies. The membranes were washed extensively with 0.1% Tween-20 in PBS, target protein bands visualized using a LiCor Odyssey infrared Imager (LiCor, Lincoln, NE).

### Statistics

Relative quantitative ratios were calculated by taking the average of the normalized Total Ion Current from all of the MS/MS spectra identifying each protein including all redundancy, charge states, and missed cleavages, and then dividing the experimental samples (AAb+, T1D and T2D cases) (numerator) by the control cases (denominator) using Scaffold. The peptide probabilities were generated using the Peptide Prophet algorithm that is embedded in Scaffold S+Q, yielding a discriminant score [22]. The data from the standard Mascot search are mapped on a histogram demarcated by discriminant scores, and Bayesian statistics are used to determine the probability that a match is correct at each discriminant score. The calculation of FDRs in Scaffold was done using the methods of Käll *et al* 2008 [25]. For all statistical analyses, P < 0.05 was considered significant.

## Results

### Comparative Proteome Analysis of Pancreas Tissues

**Fig 1:**
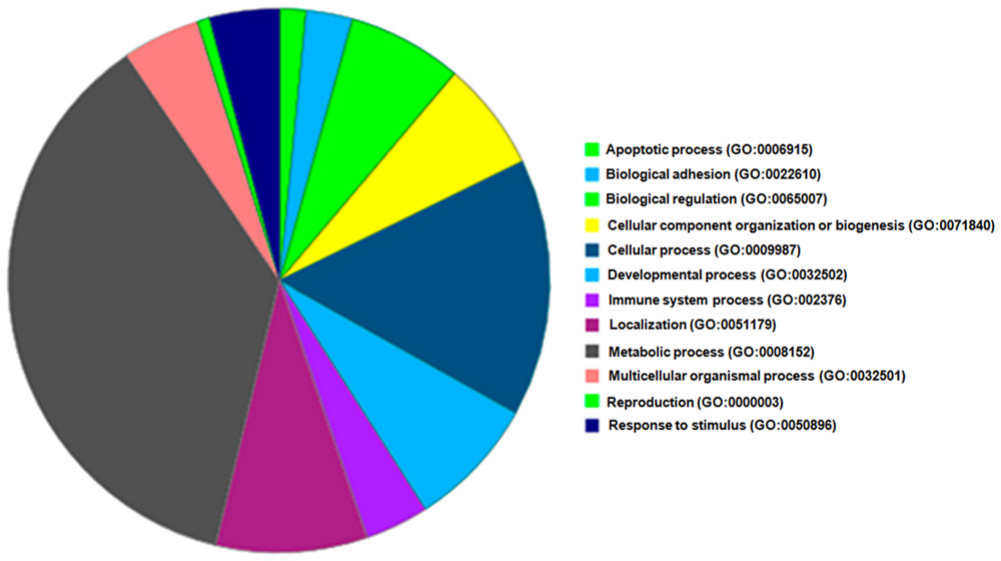
Functional categories and biological processes of all the proteins that are identified in ND, AAb+, T1D and T2D cases.

A key goal of our study was to provide, for the first time, a comprehensive comparative proteomic analysis of disease-stratified pancreata that would be useful in improving our understanding of the pathogenesis or identifying possible biomarkers of T1D. We have compared the proteomic profile of pancreas tissue from ND, AAb+, T1D, and T2D donors using a label-free proteomic approach (S1 Fig). 1,167 proteins were identified from the combined MS runs, consisting of 1,147 proteins from ND, 1,143 from AAb+, 1,149 from T1D and 1,132 proteins from T2D cases; of these, 1,085 proteins were shared among the four groups. Fig 1 summarizes the functional categories of the identified proteins. The proteins are involved in a wide range of cellular biological processes and functions, including apoptotic processes, adhesion, biological regulation, cellular component organization or biogenesis, immune system processes, localization, metabolic processes, and response to stimulus, among others. The detailed lists of all identified proteins in the groups and their biological processes are presented in S3 Table.

**Fig 2:**
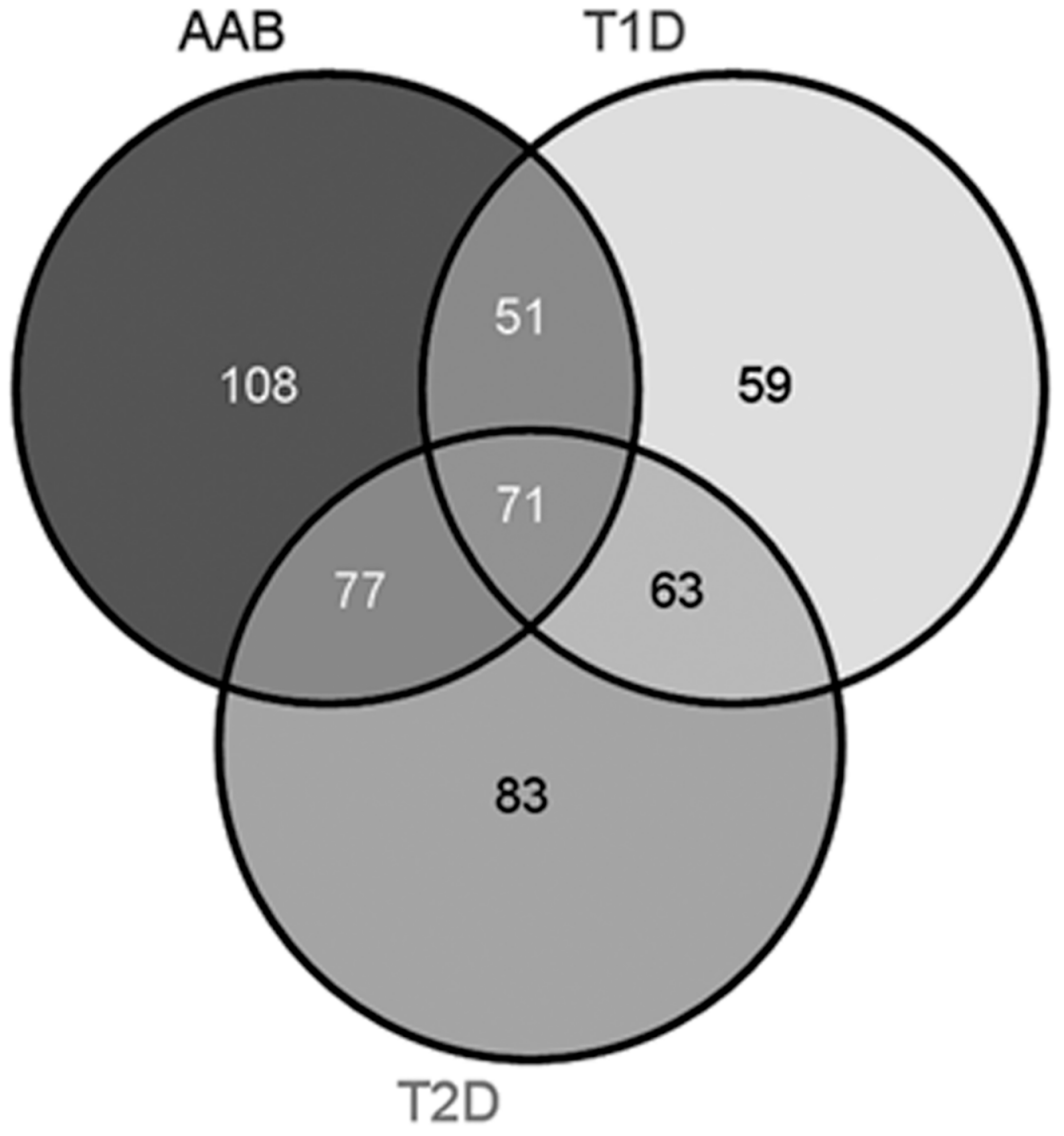
Venn diagram comparison of differentially regulated proteins in AAb+, T1D and T2D cases compared to ND. The cut-off for the threshold fold change differences were ≥ 2.0 for upregulation and ≤ 0.05 for down regulation with p- values < 0.05. The list of the differentially regulated proteins are shown in S4–S6 Tables.

**Fig 3:**
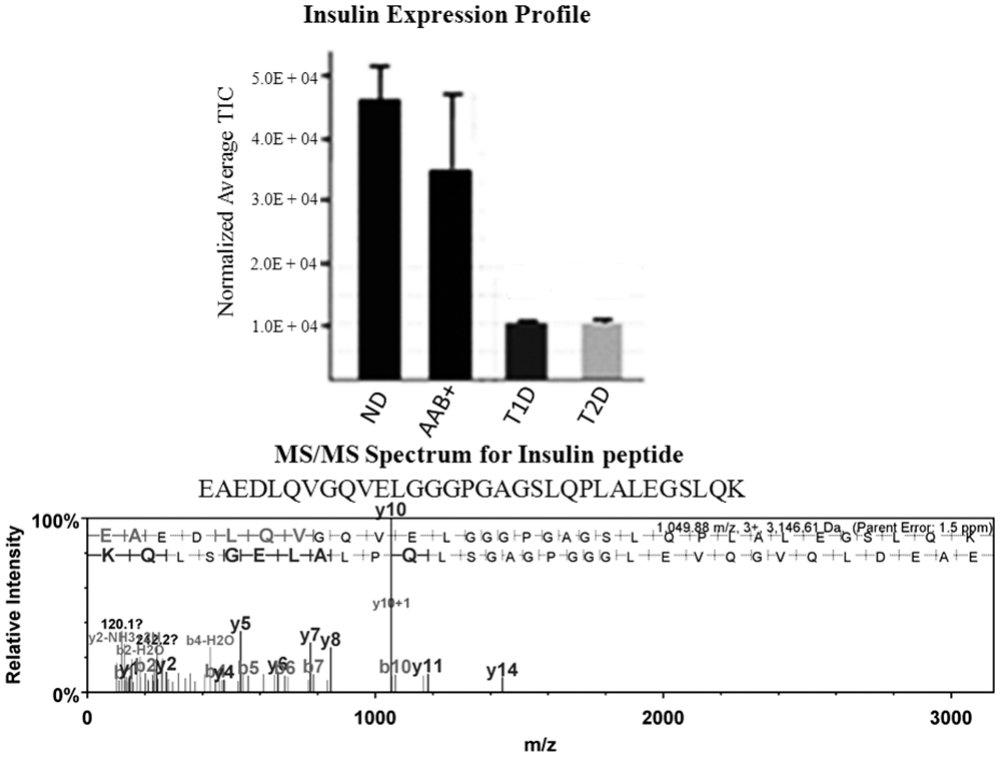
Insulin Expression Profile. Normalized average total ion chromatogram for insulin in ND, AAb+, T2D and T1D cases were determined. Insulin is significantly downregulated in the T1D and T2D cases. Student’s t-test is used for statistical analysis. All data are mean ± SEM. *P<0.05. The mass spectrum for a representative insulin peptide (m/z, 1049.88 (+3)) with sequence EAEDLQVGQVELGGGPGAGSLQP LALEGSLQK is shown in the lower panel.

Scaffold S+Q was used to analyze differential protein expression levels between the four groups. For the AAb+ cases, 307 proteins were differentially regulated compared to the no-disease cohort, with 65 upregulated and 242 downregulated proteins (S4 Table). For the T1D samples, 244 proteins were differentially expressed, with 134 upregulated and 110 downregulated, compared to the no disease controls (S5 Table). In the T2D sample cohort, 294 proteins were differentially regulated, with 95 upregulated and 199 downregulated, compared to the no disease samples (S6 Table). Fig 2 shows the comparison of the differentially expressed proteins between the no-disease samples and the other three sample cohorts. Both exocrine- and endocrine-derived proteins were identified in these analyses. Fig 3 shows the average total ion currents showing the expression profile for insulin in the four sample groups and MS/MS spectra for an insulin peptide. As expected, the abundance of insulin was significantly downregulated in T1D and T2D cases compared to control and AAb+ donors. We have previously demonstrated differential expression of insulin and other proteins using imaging mass spectrometry [29]. S2 Fig shows the same information for glucagon with identical among the four sample categories. These two proteins are endocrine derived. The expression profiles and MS/MS spectra for two exocrine derived proteins, chymotrypsin and pancreatic alpha amylase, are shown in S3 Fig Similar data for housekeeping proteins, GAPDH and beta tubulin are shown in S4 Fig. No significant differences were found in the expression profiles of the exocrine-derived chymotrypsin and pancreatic alpha amylase, or housekeeping proteins GAPDH and beta tubulin.

### Validation of Proteomics Data

To validate the above proteomics data, we analyzed the expression pattern of three proteins RegIIIα, OFLM4 and ENPP1 that are amongst the significantly upregulated proteins in T1D cases versus the ND, AAb+, and T2D cases in pooled samples from the four groups by Western blot. Glucagon and GAPDH were included as loading controls, and their abundance was identical in the four sample cohorts (S5 Fig).

### Pathway and Network Analysis Demonstrate Unique Features in Different Disease States

**Fig 4:**
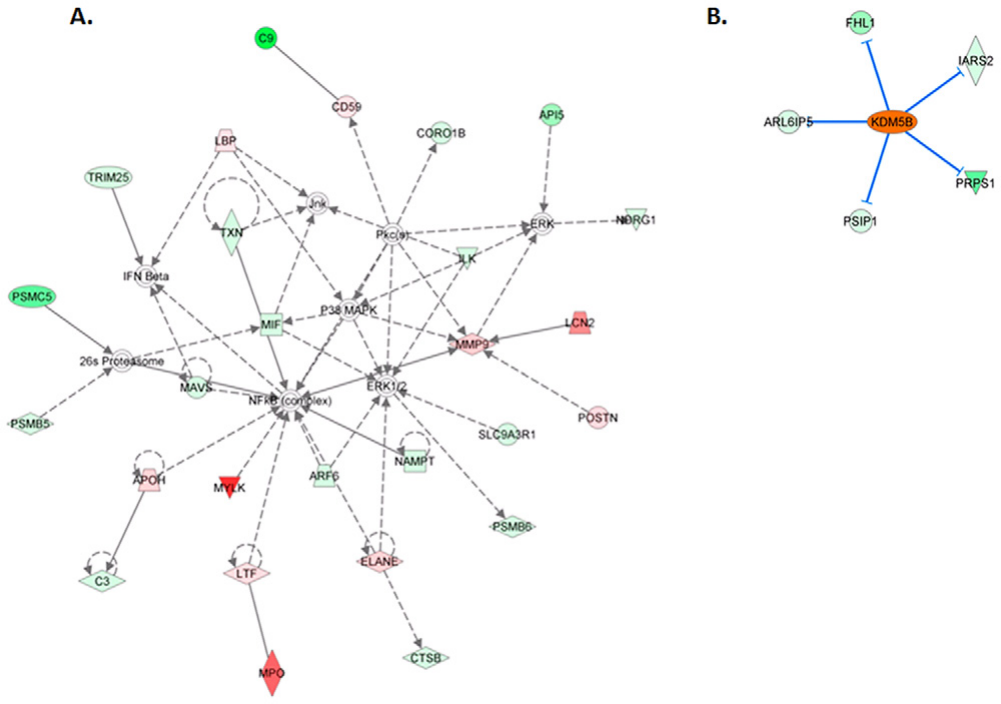
(A) Ingenuity pathway analysis showing top interaction network for differentially regulated proteins in AAb+ cases. Those highlighted with red color are upregulated genes and those with green are downregulated genes. The names of these genes are listed in S7 Table. (**B).** Ingenuity pathway analysis depicting the activated transcription factor KDM5B) in AAb+ cases when compared to controls. This leads to the inhibition of FHL1, IARS2, ARL6IP5, PSIP1 and PRPS1.

The molecular processes, molecular functions, and genetic networks in the AAb+, T1D, and T2D cases were further evaluated by analyzing differentially expressed proteins using ingenuity pathways analysis (IPA) (Ingenuity Systems, www.ingenuity.com). In the case of AAb+ cases, the top network (score = 37; 27 focus molecules) has the following top diseases and functions: Cellular Movement, Hematological System Development and Function, and Immune Cell Trafficking. Several of the differentially upregulated molecules are indirectly interconnected and involved in important components of the functional network (Fig 4A). There are potentially 4 central nodes that include NF-κB, a master regulator of the immune response, ERK1/2, P38MAPK, and MMP9. Several molecules involved in the modulation and regulation of the immune system are represented in the network, including complement proteins C3, C9, and CD59, as well as interferon beta. Other molecules of interest in the network include the mitochondrial antiviral signaling protein and macrophage migration inhibitory factor. Myeloperoxidase (MPO) is amongst the most significantly upregulated molecules in the network. MPO is a member of the heme peroxidase super-family, is an inherent cellular component of leukocytes, mainly found in macrophages and neutrophils that are released during inflammation. We performed IPA up/down stream analysis to predict the effect of gene expression changes in the AAb+ samples in terms of biological processes and disease, or cellular functions compared to ND controls. Based on the probability scores, the top ten were HNF1A, LH, FSH, CCL5, HCK, ELANE, CXCL8, NEUROG1, PDX1 and 15-LOX. HNF1-alpha is a transcription factor that has several roles in cells including apoptosis, proliferation, differentiation and transactivation. HNF1-alpha is also involved in the modulation of maturity-onset diabetes of the young [30–31]. The other transcriptional regulators include NEUROG1 and PDX1, which is a transcriptional activator of several genes, including insulin, somatostatin, glucokinase, islet amyloid polypeptide, and glucose transporter type 2. The most significantly upregulated molecule in the group is ELANE, with a 4.8 fold increase in the AAb+ group compared to ND cohorts. ELANE is an acute inflammatory response molecule produced in response to antigenic stimulus, and is a marker for positive regulation of immune responses. CCL5 and CXCL8 are also upstream regulators in the AAb+ group compared to the ND cases. Although all the preceding molecules have significant probability scores, they are not predicted to be activated in AAb+ cases compared to ND cases in our current analyses. To confirm the activation of specific pathways, IPA uses the regulation *z*-score algorithm to make predictions. The *z*-score algorithm is designed to reduce the chance that random data will generate significant predictions. The transcription regulator lysine (K)-specific demethylase 5B (KDM5B) is predicted to be activated in the AAb+ cases compared to the ND cohort. KDM5B is a transcriptional regulator of multiple molecular functions including the regulation of various dioxygenase activities. The network associated with KDM5B is shown in Fig 4B. This transcription factor leads to the inhibition of FHL1, IARS2, ARL6IP5, PSIP1 and PRPS1, some of which have been implicated in processes that are involved in the progression of different conditions leading to diabetes. For example, ARL6IP5 may play a role within the apoptotic network activated in pancreatic β cells during insulitis [32].

Similar analyses were done using proteins that are differentially regulated between T1D cases compared to the ND cases. For the T1D cases, the top network (score = 30; 25 focus molecules) has the following top diseases and functions: Inflammatory Response, Inflammatory Disease, Cell Death and Survival, Cell to Cell Signaling and Interaction (S6 Fig). The top ten upstream regulators based on their probability scores include POU2F1, PACS2, ITGB1, P2RY2, PSMB5, ACTN4, GFI1, FUBF1, RFXANK and CIITA. POU class 2 homeobox 1 (POU2F1), also known as octamer-binding transcription factor-1 (OCT-1), is a ubiquitous transcription factor that helps regulate genes related to inflammation and cell cycles. Other transcription regulators include PACS2 (apoptosis) and GFI1 (transcriptional repressor nuclear zinc finger protein). RFXANK (primary immunodeficiency signaling) and CIITA (class II major histocompatibility complex transactivator involved in Antigen Presentation Pathway, Primary Immunodeficiency Signaling and TREM1 Signaling) were other high scoring molecules. Additionally, beta 5 integrin has the second highest probability score in the upstream regulator list in T1D compared to ND donors. Integrin beta 5 is involved in the following pathways: Caveolar-mediated Endocytosis Signaling, Clathrin-mediated Endocytosis Signaling, IL-8 Signaling, ILK Signaling, Integrin Signaling, NF-κB Activation by Viruses, Paxillin Signaling, PPARα/RXRα Activation, and Virus Entry via Endocytic Pathways. The proteins that are differentially regulated between T2D cases and the ND cases were also analyzed using the same strategy previously used for AAb+ and T1D cases. The results are summarized in S7 Fig.

### Pathway and Network Analysis of Uniquely Upregulated Proteins in the Sample Cohorts

**Fig 5:**
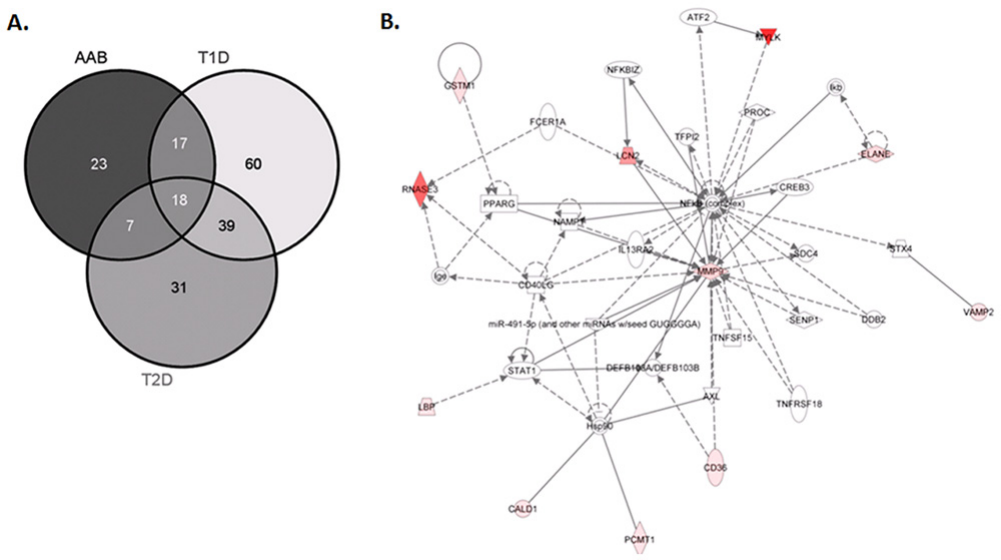
(A) Venn diagram comparison of uniquely upregulated proteins in AAb+, T1D and T2D cases compared to ND. The cut-off for the threshold fold change differences were ≥ 2.0 for upregulation with p- values < 0.05. **(B).** Ingenuity pathway analysis showing top interaction network for uniquely upregulated proteins in AAb+ cases. Those highlighted with red color are upregulated genes and those with green are downregulated genes. The names of these genes are listed in S8 Table.

AAb+, T1D and T2D cases were analyzed for proteins that were uniquely and significantly upregulated compared to ND cases. These proteins are most likely to be involved in the progression, development and pathogenesis of the two diseases, and are potential markers for diagnosis and prognosis of the morbidities. AAb+, T1D, and T2D cases were compared for overlap in the expression of upregulated proteins (Fig 5A). This comparison allowed us to determine that 23 proteins are uniquely upregulated in the AAb+ cases, 60 proteins in the T1D cases, and 31 proteins in the T2D cases.

IPA network analysis was performed to identify potential markers that are differentially upregulated in only in AAb+, T1D, and/or T2D versus the ND cases. For the AAb+ cases, the central nodes include the NF-κB complex, MMP9, STAT1, and Hsp90 (Fig 5B). Consistent with the phenotype of the AAb+ cases, NF-κB is a master regulator of the immune response, whereas MMP9 has been implicated matrix remodeling that may be associated with disease pathogenesis. STAT1, the signaling molecule downstream of IFN-γ, is known to be important for cell viability in response to different cell stimuli and pathogens, including viruses. For the T1D cases, the central nodes include TGFB1, IL1B, and CXCL8. TGFB1 is reported to have a role in regulating beta cell growth in early life, whereas IL1B and CXCL8 are mediators of islet inflammation and dysfunction (S8 Fig). For the T2D cases, the central nodes are TNF and TGM2 (S9 Fig). Defects in TGM2 gene are associated with early onset of Type 2 diabetes, and TNF-alpha has been implicated as a causative factor in obesity-associated insulin resistance and the pathogenesis of type 2 diabetes.

### Potential Biomarker Roles of Uniquely Upregulated Proteins in the Sample Cohorts

**Fig 6:**
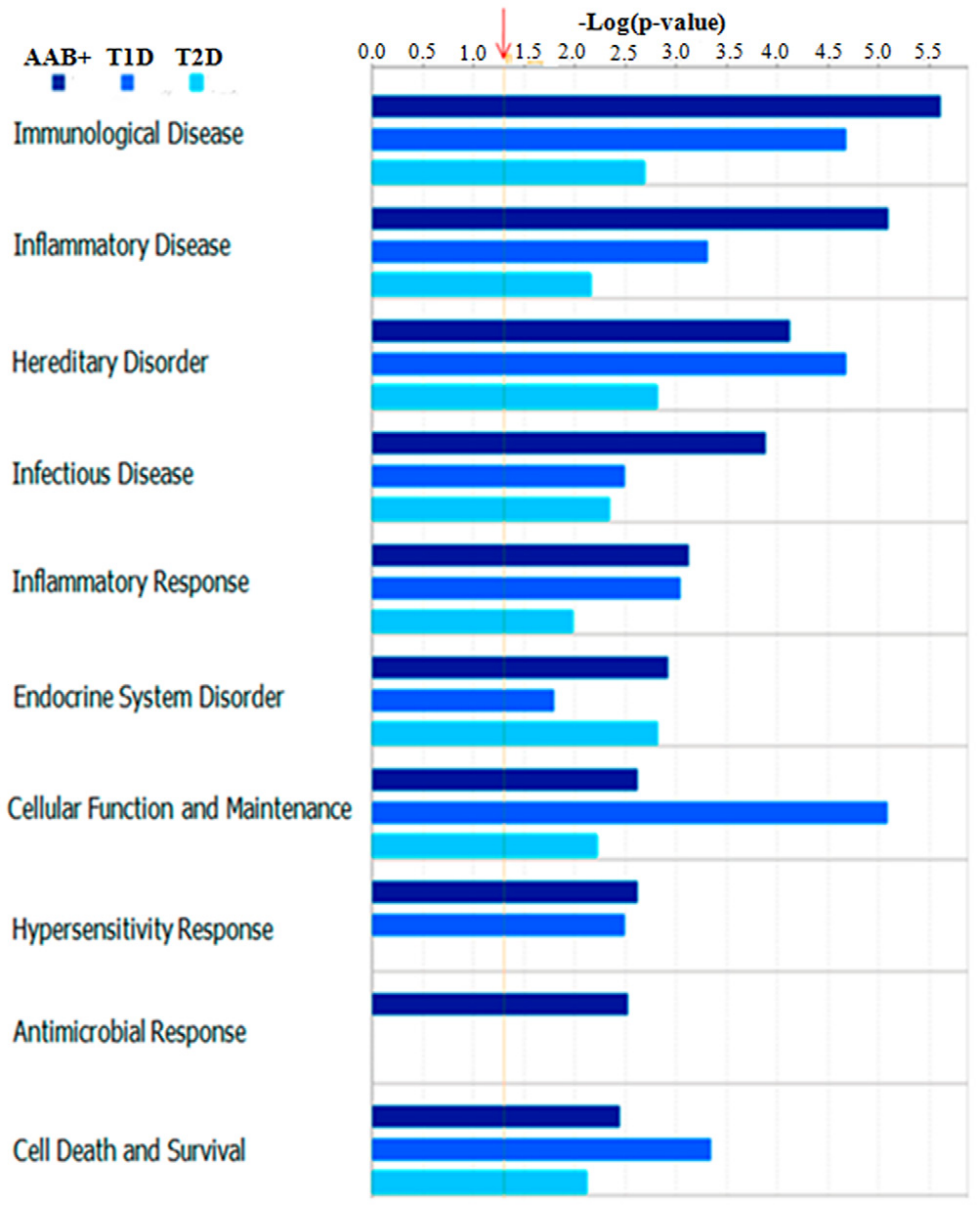
Comparative IPA analysis of proteins that are uniquely upregulated in AAb+, T1D and T2D cases. There is a unique upregulation of specific pathways, diseases and biofunctions in the different sample categories.

**Fig 7:**
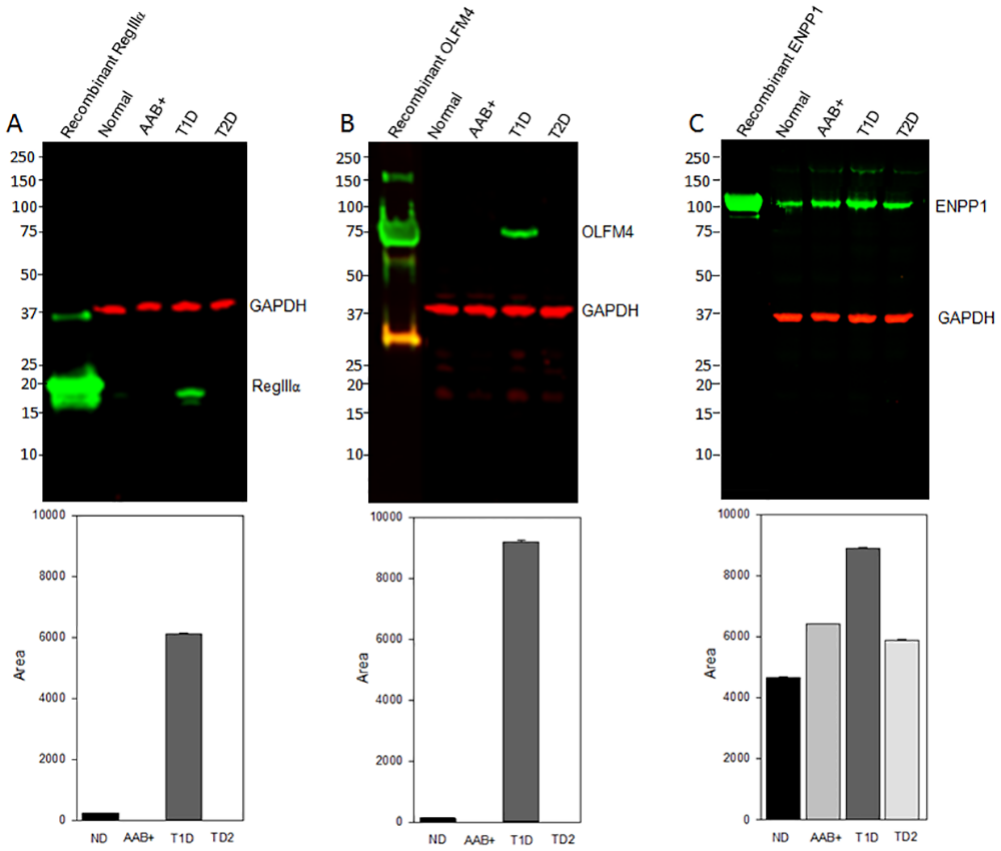
Validation of differentially upregulated of specific proteins in pancreas tissue lysates. Normalized total protein lysates from pooled normal, Aab+, T1D and T2D samples were subjected Western blot analysis to detect for REGIIIα/γ (Panel A), Olfactomedin 4 (Panel B), and ENPP1 (Panel C),, . GAPDH detection was included as a loading control. ImageJ analysis was used to confirm the expression of the three proteins after normalization using GAPDH values. The bar graphs represent data from triplicate analyses.

Uniquely upregulated proteins in AAb+, T1D, and T2D cases as compared to the ND cases may serve as diagnostic and prognostic markers of the diseases. Comparative IPA analysis of the 3 cohorts revealed the unique upregulation of specific pathways, diseases, and biofunctions that correlate specifically do each disease state, with p values that are significantly higher than the set threshold (1.3-log (p-value)) (Fig 6). It is important to note that the antimicrobial response pathway is uniquely upregulated in the AAb+ cases, and not in T1D or T2D cases. This is consistent with the hypothesis that postulates the early involvement of infectious pathogens in the etiology of T1D. To further demonstrate and confirm the upregulation of specific proteins, we have used Western Blotting to validate the exclusive upregulation of Olfactomedin 4, ENPP1, and REGIIIα/γ in T1D cases. Most importantly, strong bands for Olfactomedin4 and REGIIIα/γ were almost exclusively detected in T1D samples (Fig 7). The signal intensity observed between the different bands is significantly different when compared by densitometry using ImageJ in triplicate experiments. Therefore, the protein panels identified in the present study are potential markers for disease, and also potential therapeutic targets.

## Discussion

This is the first comprehensive study to integrate high-resolution, high mass accuracy mass spectrometry and proteomic technologies for an unbiased discovery and verification of pancreas tissue-specific proteomic changes in stratified disease cases comparing T1D patients with other groups. Sixty tissue proteins with functional relevance to T1D were found to be significantly upregulated compared to ND, AAb+, and T2D cases. From a functional point of view, these proteins are likely relevant to T1D since they are implicated in inflammation, metabolic regulation, and autoimmunity. All of these pathways have been linked to T1D pathogenesis, and could serve as protein biomarkers or offer new insight into the pathogenesis of T1D versus T2D. Our data demonstrate differential expression of both exocrine- and endocrine-derived proteins, with the potential activation of different pathways and upregulation of unique molecules in AAb+, T1D, and T2D cases when compared to controls. These results support the emerging data that propose a role of both exocrine and endocrine pancreas tissue in the development of T1D and T2D [33–34].

Among the proteins that are significantly over-expressed in T1D pancreas, but not in the other three groups is Olfactomedin 4 (OLFM4, also known as hGC-1, GW112). The glycoprotein OLFM4 is upregulated in inflammatory bowel diseases and *Helicobacter pylori* infected patients [35]. Recent studies of OLFM4 knock-out mice demonstrated that OLFM4 exerts considerable influence on the host defense against *H*. *pylori* infection, acting through NOD1- and NOD2-mediated NF-kappa B activation and subsequent cytokine and chemokine production. This, in turn, inhibits host immune responses and contributes to persistence of *H*. *pylori* colonization in the OLFM4 KO mice [35]. The role of OLFM4 is interesting in view of the hypothesis that enteroviruses may trigger T1D, and these microorganisms may use the same strategy to evade immune responses in the pancreas in T1D.

Ectonucleotide pyrophosphatase (ENPP1) negatively modulates insulin receptor activation and plays a role in insulin signaling, insulin secretion, and glucose metabolism [36–37]. Recent studies have demonstrated that ENPP1 affects both the function and survival of pancreatic beta cells, thus representing a strong pathogenic factor predisposing to insulin resistance, defective insulin secretion, and impaired glucose metabolism [38]. Polymorphisms in the ENPP1 gene have also been associated with an increased risk of end-stage renal disease early in the course of T1D [39].

The loss and dysfunction of pancreatic beta cells is a crucial factor in diabetes progression. The functional recovery, and hopefully regeneration, of beta cells is considered an important therapeutic goal in T1D. Our results demonstrate the exclusive overexpression of regenerating islet-derived protein (Reg) III α in T1D cases as compared to the other three groups. These data are consistent with the work of Choi et al., [40], who used subtractive hybridization and DNA sequencing of whole pancreas to demonstrate that Reg3α changes the expression level of islet marker genes, including NEUROD, NKX2.2, PAX4, and PAX6. Furthermore, Reg expression may serve as a biomarker for iron-related pancreatic stress, as iron changes may contribute to diabetes development through up-regulation of Alox15 [41]. Overall, RegIIIα may represent a novel therapeutic target to modulate beta cell function, survival and regeneration.

In conclusion, the present proteomic analysis provides identification of proteins that are uniquely upregulated in pre-diabetic and diabetic states, and can distinguish T1D from T2D in human tissue. Such changes are likely independent of hyperglycemia, as they were not observed in T2D cases, and are more likely linked to the disease pathogenesis through inflammatory, immune, and beta cell dysfunction pathways. The results are significant since they provide areas for follow-up studies evaluating proteins in biological fluids or in laser captured islets aimed at the implementation of new biomarkers for the early detection of T1D and therapeutic targets against the disease. This study will provide an additional perspective to previous studies evaluating the proteomics of cultured islets [14].

## Supporting Information

S1 Fig Schematic diagram of experimental workflow. (TIF)

S2 Fig Normalized average total ion currents for glucagon in ND, AAb+, T2D and T1D cases. There are no significant differences in the expression of glucagon in the four groups. Student’s t-test is used for statistical analysis. All data are mean ± SEM. P<0.05. The mass spectrum for a representative glucagon peptide (m/z, 801.91 (2+)) with sequence DFPEEVAIVEELGR is shown in the lower panel.
(TIF)

S3 Fig (A) Normalized average total ion currents for chymotrypsin in ND, AAb+, T2D and T1D cases. There are no significant differences in the expression of chymotrypsin in the four groups. Student’s t-test is used for statistical analysis. All data are mean ± SEM. P<0.05. The mass spectrum for a representative chymotrypsin peptide (m/z, 669.34 (2+)) with sequence VSAIYIDWINEK is shown in the lower panel. (B). Normalized average total ion currents for pancreatic α amylase in ND, AAb+, T2D and T1D cases. There are no significant differences in the expression of pancreatic α amylase in the four groups. Student’s t-test is used for statistical analysis. All data are mean ± SEM. P<0.05. The mass spectrum for a representative pancreatic α amylase peptide (m/z, 669.34 (2+)) with sequence TSIVHLFEWR is shown in the lower panel.
(TIF)

S4 Fig (A). Normalized average total ion currents for GAPDH in ND, AAb+, T2D and T1D cases. There are no significant differences in the expression of GAPDH in the four groups. Student’s t-test is used for statistical analysis. All data are mean ± SEM. P<0.05. The mass spectrum for a representative GAPDH peptide (m/z, 801.91 (2+)) with sequence LVINGNPITIFQER is shown in the lower panel. (B). Normalized average total ion currentss for tubulin beta in ND, AAb+, T2D and T1D cases. There are no significant differences in the expression of tubulin beta in the four groups. Student’s t-test is used for statistical analysis. All data are mean ± SEM. P<0.05. The mass spectrum for a representative tubulin beta peptide (m/z, 801.42 (2+)) with sequence ALVDLEPGTMDSVR is shown in the lower panel.
(TIF)

S5 Fig Validation of the expression of endocrine protein and housekeeping proteins in pooled pancreas tissue lysates. Normalized total protein lysates from pooled normal, AAb+, T1D and T2D samples were subjected Western blot analysis to detect for glucagon an endocrine protein and GAPDH was used as a loading control.
(TIF)

S6 Fig Ingenuity pathway analysis showing top interaction network for differentially regulated proteins in T1D cases. Those highlighted with red color are upregulated genes and those with green are downregulated genes. The names of these genes are listed in S9 Table.
(TIF)

S7 Fig Ingenuity pathway analysis showing top interaction network for differentially regulated proteins in T2D cases. Those highlighted with red color are upregulated genes and those with green are downregulated genes. The names of these genes are listed in S10 Table. The proteins that are differentially regulated between T2D cases and the ND cases were also analyzed using the same strategy previously used for AAb+ and T1D cases. For the T2D cases, the top network (score = 19; 11 focus molecules, has four potential central nodes, including NF-κB, Integrin beta 1, ERK1/2 and FN1. The top ten upstream regulators based on probability scores include the following: SF1, LGALS1, JAK, AMPK, SUPT16H, SSRP1, PACS2, ACOT8, RYK, and PPAP28. SF1 and SUPT16H are transcriptional regulators, JAK and RYK are kinases, and PPAP28 is a phosphatase. It is important to note that JAK is identified in the T2D cases, but not in AAb+ and T1D cases.
(TIF)

S8 Fig Ingenuity pathway analysis showing top interaction network for uniquely upregulated proteins in T1D cases. Those highlighted with red color are upregulated genes and those with green are downregulated genes. The names of these genes are listed in S11 Table.
(TIF)

S9 Fig Ingenuity pathway analysis showing top interaction network for uniquely upregulated proteins in T2D cases. Those highlighted with red color are upregulated genes and those with green are downregulated genes. The names of these genes are listed in S12 Table.
(TIF)

S1 File Supplemental Methods File. (PDF)

S1 Table Donor phenotype and pancreas tissue samples used in the study. (PDF)

S2 Table Antibodies and proteins used in the study. (PDF)

S3 Table List of the identified proteins and their biological processes. (XLSX)

S4 Table List of differentially regulated proteins between no disease (ND) and autoantibody positive (AAb+) cases. (PDF)

S5 Table List of differentially regulated proteins between no disease (ND) and type 1 diabetes (T1D) cases. (PDF)

S6 Table List of differentially regulated proteins between no disease (ND) and type 2 diabetes (T2D) cases. (PDF)

S7 Table List of genes represented in the network for differentially expressed proteins in AAb+ versus ND in Fig 4A. (PDF)

S8 Table List of genes represented in the network for differentially expressed proteins in T1D versus ND in Fig 5A. (PDF)

S9 Table List of genes represented in the network for differentially expressed proteins in T2D versus ND in S6 Fig. (PDF)

S10 Table List of genes represented in the network for uniquely upregulated proteins in AAb+ versus ND in S7 Fig. (PDF)

S11 Table List of genes represented in the network for uniquely upregulated proteins in T1D versus ND in S8 Fig. (PDF)

S12 Table List of genes represented in the network for uniquely upregulated proteins in T2D versus ND in S9 Fig. (PDF)
