# Supplementary figures and images for: Proteomic Analysis of Disease Stratified Human Pancreas Tissue Indicates Unique Signature of Type 1 Diabetes
Source: PLoS One. 2015 Aug 24;10(8):e0135663. doi: 10.1371/journal.pone.0135663 (PMC4547762; doi:10.1371/journal.pone.0135663)

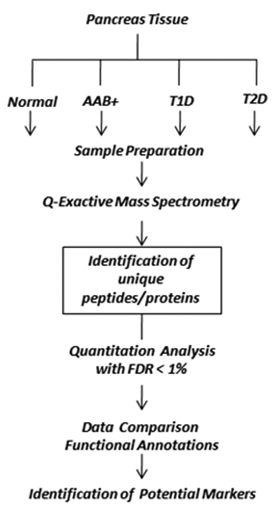

Supplement: S1 Fig — (TIF) [file pone.0135663.s001.tif]

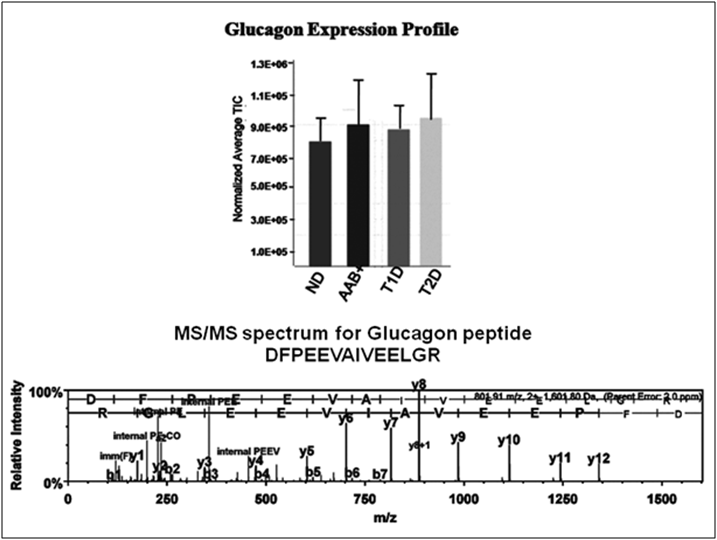

Supplement: S2 Fig — There are no significant differences in the expression of glucagon in the four groups. Student’s t-test is used for statistical analysis. All data are mean ± SEM. P<0.05. The mass spectrum for a representative glucagon peptide (m/z, 801.91 (2+)) with sequence DFPEEVAIVEELGR is shown in the lower panel. (TIF) [file pone.0135663.s002.tif]

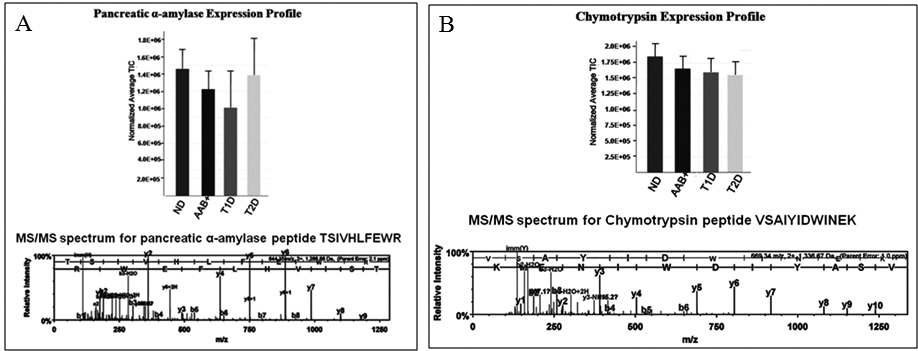

Supplement: S3 Fig — There are no significant differences in the expression of chymotrypsin in the four groups. Student’s t-test is used for statistical analysis. All data are mean ± SEM. P<0.05. The mass spectrum for a representative chymotrypsin peptide (m/z, 669.34 (2+)) with sequence VSAIYIDWINEK is shown in the lower panel. (B). Normalized average total ion currents for pancreatic α amylase in ND, AAb+, T2D and T1D cases. There are no significant differences in the expression of pancreatic α amylase in the four groups. Student’s t-test is used for statistical analysis. All data are mean ± SEM. P<0.05. The mass spectrum for a representative pancreatic α amylase peptide (m/z, 669.34 (2+)) with sequence TSIVHLFEWR is shown in the lower panel. (TIF) [file pone.0135663.s003.tif]

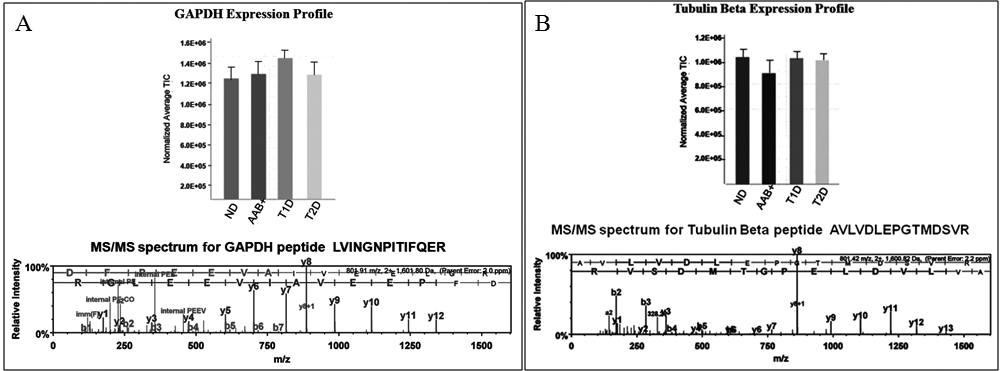

Supplement: S4 Fig — There are no significant differences in the expression of GAPDH in the four groups. Student’s t-test is used for statistical analysis. All data are mean ± SEM. P<0.05. The mass spectrum for a representative GAPDH peptide (m/z, 801.91 (2+)) with sequence LVINGNPITIFQER is shown in the lower panel. (B). Normalized average total ion currentss for tubulin beta in ND, AAb+, T2D and T1D cases. There are no significant differences in the expression of tubulin beta in the four groups. Student’s t-test is used for statistical analysis. All data are mean ± SEM. P<0.05. The mass spectrum for a representative tubulin beta peptide (m/z, 801.42 (2+)) with sequence ALVDLEPGTMDSVR is shown in the lower panel. (TIF) [file pone.0135663.s004.tif]

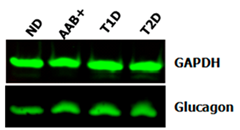

Supplement: S5 Fig — Normalized total protein lysates from pooled normal, AAb+, T1D and T2D samples were subjected Western blot analysis to detect for glucagon an endocrine protein and GAPDH was used as a loading control. (TIF) [file pone.0135663.s005.tif]

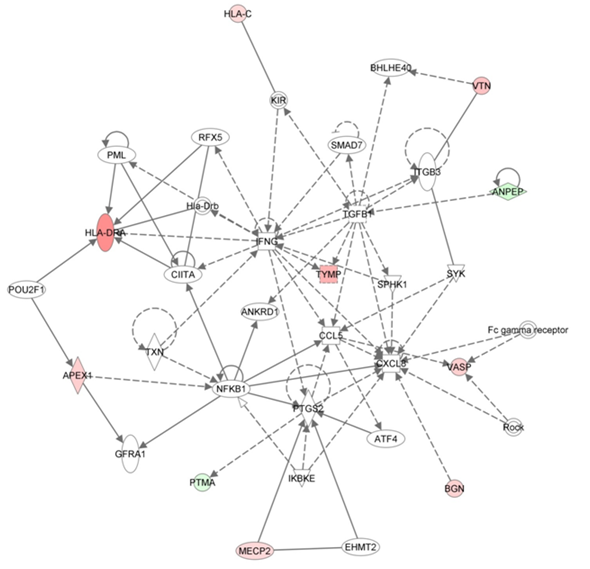

Supplement: S6 Fig — Those highlighted with red color are upregulated genes and those with green are downregulated genes. The names of these genes are listed in S9 Table. (TIF) [file pone.0135663.s006.tif]

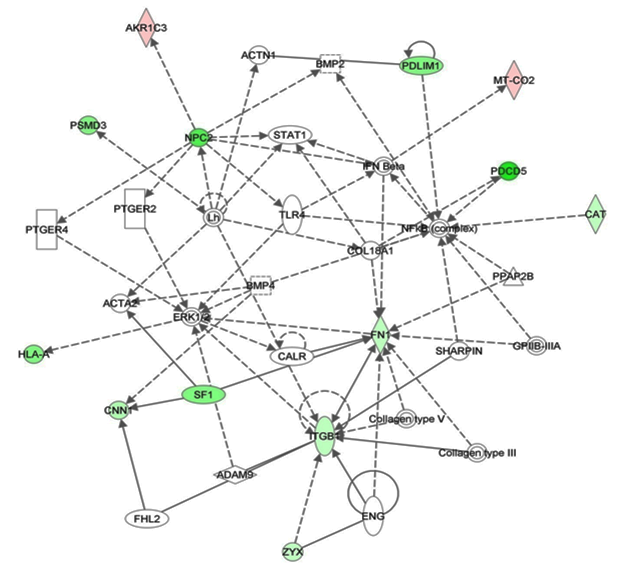

Supplement: S7 Fig — Those highlighted with red color are upregulated genes and those with green are downregulated genes. The names of these genes are listed in S10 Table. The proteins that are differentially regulated between T2D cases and the ND cases were also analyzed using the same strategy previously used for AAb+ and T1D cases. For the T2D cases, the top network (score = 19; 11 focus molecules, has four potential central nodes, including NF-κB, Integrin beta 1, ERK1/2 and FN1. The top ten upstream regulators based on probability scores include the following: SF1, LGALS1, JAK, AMPK, SUPT16H, SSRP1, PACS2, ACOT8, RYK, and PPAP28. SF1 and SUPT16H are transcriptional regulators, JAK and RYK are kinases, and PPAP28 is a phosphatase. It is important to note that JAK is identified in the T2D cases, but not in AAb+ and T1D cases. (TIF) [file pone.0135663.s007.tif]

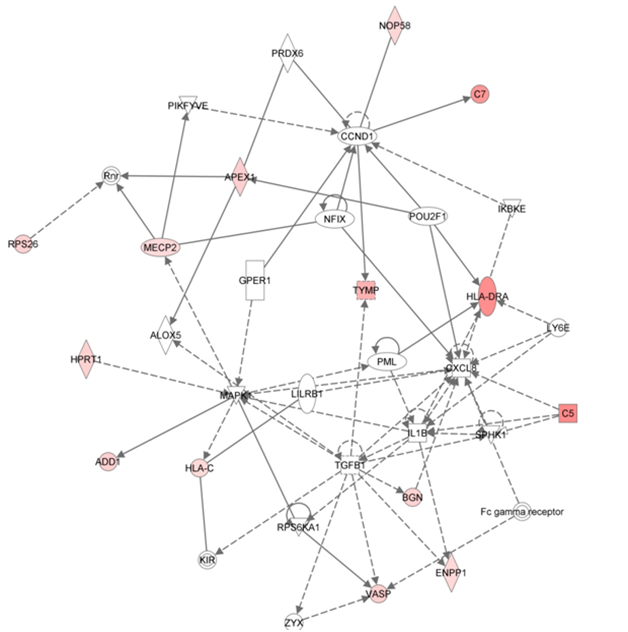

Supplement: S8 Fig — Those highlighted with red color are upregulated genes and those with green are downregulated genes. The names of these genes are listed in S11 Table. (TIF) [file pone.0135663.s008.tif]

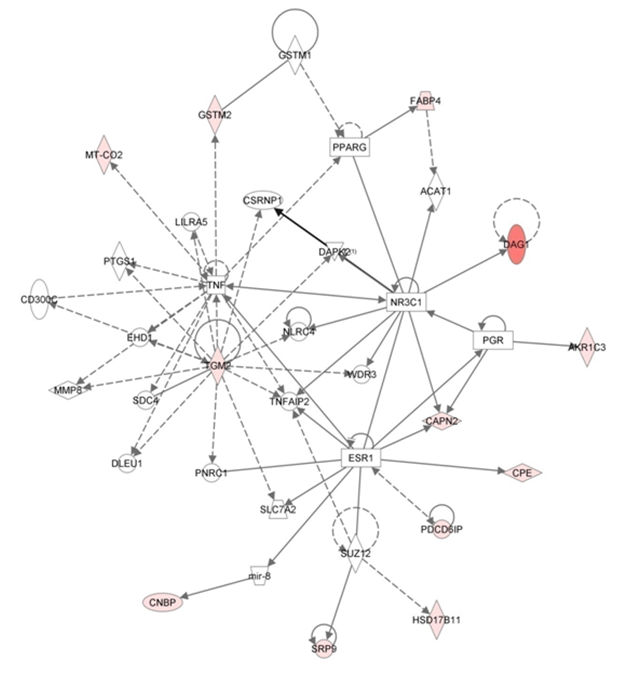

Supplement: S9 Fig — Those highlighted with red color are upregulated genes and those with green are downregulated genes. The names of these genes are listed in S12 Table. (TIF) [file pone.0135663.s009.tif]
