# Supplementary material for: Proteomic Analysis of Disease Stratified Human Pancreas Tissue Indicates Unique Signature of Type 1 Diabetes
Source: PLoS One. 2015 Aug 24;10(8):e0135663. doi: 10.1371/journal.pone.0135663 (PMC4547762; doi:10.1371/journal.pone.0135663)
Supplement: S1 File — (PDF) [file pone.0135663.s010.pdf]

Supplemental Information and Methods

nPOD cases classification

nPOD classifies patients using these criteria: ND did not have diabetes and tested negative for T1D-associated autoantibodies, in the absence of rare disease or other dramatic co-morbidity; AAb+ donors did not have diabetes, but their serum or plasma tested positive for at least one T1D-related autoantibody [7]; donors with T1D had a clinical history of diabetes and their medical history, donor questionnaire, and additional laboratory data (AAb, C-peptide, HLA) confirmed the diagnosis; donors with T2D had a clinical history of T2D and tested negative for autoantibodies.

Sample Preparation and Mass Spectrometry Analysis and Protein Quantitation

Snap frozen pancreas tissue samples (5 from each group) were lysed in 50% TFE, 50 mM triethyl ammonium bicarbonate (TEAB) pH 8.0, and incubated for 30 min at 4°C. The mixtures were homogenized before sonication using 20 s bursts, followed by ice cooling (20 s), 5 times. The samples were heated at 60°C for 30 minutes, and sonicated again prior to centrifugation for 15 min at 10, 000 x *g* to remove particulate matter. The protein concentration was determined by BCA protein assay. Normalized protein concentrations from each sample were reduced, alkylated, and trypsin digested for 18 hrs at 37°C. The generated peptides were acidified using formic acid, and centrifuged at 20,000 x *g* for 10 mins. The supernatant was desalted using C18 columns and the eluted peptides dried *in vacuo* prior to further analysis. Tryptic peptides were solubilized in normalized volumes of 0.1% formic acid (FA)/H_2O and the concentration of the digested peptides were determined using the Micro BCA assay. The final concentration of the samples was adjusted to 0.5µg/µl the same buffer. Identical concentrations (1µg) of the peptides were analyzed on a Q-Exactive Orbitrap mass spectrometer coupled to an Easy NanoLC-1000 system under the following conditions: LC solvents: buffer A, 0.1% formic acid/water; buffer B,

0.1% formic/acetonitrile; column, Thermo Scientific™ EASY-Spray™, 75µm x 150mm C18 column; flow rate 200 nl/min; LC separation, stepped linear gradient, 5% to 30% B in the first 45 minutes, followed by 80% B in the next 5 minutes and holding at 80% B for 10 minutes. The MS data was acquired using data-dependent acquisition with following optimized settings: dynamic exclusion (DE=1); top 12 higher energy collision induced dissociation (HCD); resolving power set at 70,000 for the full MS scan and 17,500 for the MS/MS scan at m/z 200. For each individual sample triplicate LC/ESI-MS/MS analyses were performed. A label free average total ion chromatogram strategy was used for the quantitation of protein abundance using Scaffold™ Q + S (Proteome Software Inc., Portland, OR). Relative protein quantitation was achieved by the confident validation and quantitation of the search results using peptide intensity data computed by the identification software. This was achieved for each sample by subtracting the mean log intensity for the sample before adding back the mean of all log intensities globally. After data normalization across all the 20 biological samples and the two technical replicate LC-MS runs, the average total ion current (TIC) was calculated for each protein and each donor cohort (ND, AAb+, T1D and T2D). By using the average TIC, rather than the sum of TIC, the sampling bias caused by different protein molecular weights since larger proteins generate more tryptic peptides than smaller proteins is eliminated [24]. The mean normalized total ion current intensities for the peptides for each protein in each sample category were used for fold-change calculations using Scaffold™. To calculate the relative ratios for proteins that were not detected in any of the four disease categories a background intensity level of 10^3 was used. Proteins were considered to be differentially regulated between the sample cohorts if they complied with the following parameters: FDR <0.05 and fold change (FC) ≥ 2.0 , with p- values < 0.05 based on the Scaffold™ algorithm.
