# Supplementary material for: Proteomic Analysis of Disease Stratified Human Pancreas Tissue Indicates Unique Signature of Type 1 Diabetes
Source: PLoS One. 2015 Aug 24;10(8):e0135663. doi: 10.1371/journal.pone.0135663 (PMC4547762; doi:10.1371/journal.pone.0135663)
Supplement: S1 Table — (PDF) [file pone.0135663.s011.pdf]

S1 Table. Donor phenotype and pancreas tissue samples used in the study. [a] serum not available. [NK] not known.

nPOD CaseID	Donor Phenotype	AutoAb (RIA) Results	Age (Years)	Gender	Duration of Disease (Years)	C-Peptide (ng/ml)	BMI	Islet Insulin Phenotype
6095	Normal	ND[a]	40	M	-	ND[a]	35.5	Ins+ Islets
6096	Normal	Negative	16	F	-	2.97	18.8	Ins+ Islets
6104	Normal	Negative	41	M	-	20.55	20.5	Ins+ Islets
6024	Normal	Negative	24	M	-	3.52	27.8	Ins+ Islets
6140	Normal	Negative	38	M	-	11.1	21.7	Ins+ Islets
6170	AAb+	GADA	34.4	F	-	5.43	26.3	Ins+ Islets
6197	AAb+	GADA, IA-2A	22	M	-	17.48	28.2	Ins+ Islets
6151	AAb+	GADA	30	M	-	5.49	24.2	Ins+ Islets
6158	AAb+	mIAA, GADA	40.3	M	-	0.51	29.7	Ins+ Islets
6167	AAb+	IA-2A, ZnT8A	37	M	-	5.43	26.3	Ins+ Islets
6180	T1D	GADA, mIAA, IA-2A, ZnT8A	27.1	M	11	<0.05	25.9	Ins+ Islets rare
6195	T1D	GADA, mIAA, IA-2A, ZnT8A	19.2	M	5	<0.05	23.7	Ins+ Islets rare
6211	T1D	GADA, mIAA, IA-2A, ZnT8A	24	F	2	<0.05	24.4	Ins+ Islets reduced
6212	T1D	mIAA	20	M	5	<0.05	29.1	Ins+ Islets reduced
6198	T1D	GADA, mIAA, IA-2A, ZnT8A	22	F	3	<0.05	23.1	Ins+ Islets
6028	T2D	negative	33.2	M	17	22.4	30.2	Ins+ Islets
6249	T2D	mIAA	45	F	15	4.17	32.2	Ins+ Islets
6252	T2D	negative	20	M	NK	0.14	37.8	Ins+ Islets
6127	T2D	mIAA	44.2	F	10	<0.08	30.4	Ins+ Islets reduced
6142	T2D	mIAA	29.8	F	14	0.19	34.4	Ins+ Islets reduced
