# Supplementary material for: Proteomic Analysis of Disease Stratified Human Pancreas Tissue Indicates Unique Signature of Type 1 Diabetes
Source: PLoS One. 2015 Aug 24;10(8):e0135663. doi: 10.1371/journal.pone.0135663 (PMC4547762; doi:10.1371/journal.pone.0135663)
Supplement: S2 Table — (PDF) [file pone.0135663.s012.pdf]

S2 Table. Antibodies and proteins used in the study.

Antigen/Antibody	Species	Cat.	Dilution	Vendor
Anti-GAPDH	Rabbit polyclonal	sc-25778	1:1000	Santa Cruz Biotechnology, CA, USA
Anti-GAPDH	Goat polyclonal	sc-20357	1:1000	Santa Cruz Biotechnology, CA, USA
Anti-Glucagon	Goat polyclonal	sc-7780	1:200	Santa Cruz Biotechnology, CA, USA
Anti- Ectonucleotide pyrophosphatase/ phosphodiesterase 1	Rabbit polyclonal	PA5-1709	1:200	Thermo Fisher Scientific, Rockford, IL, USA
Anti-Olfactomedin-4	Rabbit polyclonal	sc-134854	1:200	Santa Cruz Biotechnology, CA, USA
Anti-Reg IIIα/γ	Rabbit polyclonal	sc-84276	1:200	Santa Cruz Biotechnology, CA, USA
Secondary IRDye680	Donkey	926-68072	1:20,000	Li-COR Biosciences, Lincoln, PA,USA
Secondary IRDye800	Donkey	926-3221	1:20,000	Li-COR Biosciences, Lincoln, PA,USA
RegIIIα full length protein	Human	Ab63303	N/A	Abcam, MA, USA
Ectonucleotide pyrophosphatase/ phosphodiesterase 1	Human	6136-EN-010	N/A	R&D Systems Inc, MN, USA
Olfactomedin-4 Full Length Protein	Human	Ab167764	NA	Abcam, MA, USA
