# Supplementary material for: Proteomic Analysis of Disease Stratified Human Pancreas Tissue Indicates Unique Signature of Type 1 Diabetes
Source: PLoS One. 2015 Aug 24;10(8):e0135663. doi: 10.1371/journal.pone.0135663 (PMC4547762; doi:10.1371/journal.pone.0135663)
Supplement: S4 Table — (PDF) [file pone.0135663.s014.pdf]

S4 Table. List of differentially regulated proteins between no disease (ND) and autoantibody positive (AAb+) cases. Only proteins with a change of 2 fold or more are included in this list. P*<0.05.

Protein Description	Accession Number	Fold Change* AAb+ vs ND
Asporin ASPN	ASPN_HUMAN	6.3
Astrocytic phosphoprotein	PEA15_HUMAN	-10
ATP synthase subunit b, mitochondrial	AT5F1_HUMAN	-2.5
ATP synthase subunit f, mitochondrial	ATPK_HUMAN	-5
ATPase inhibitor, mitochondrial	ATIF1_HUMAN	-5
ATP-dependent RNA helicase	DDX3X_HUMAN	-2
ATP-dependent RNA helicase	DDX1_HUMAN	-3.3
B-cell receptor-associated protein 31	BAP31_HUMAN	-2.5
Beta-2-glycoprotein 1	APOH_HUMAN	4.6
Bifunctional ATP-dependent dihydroxyacetone kinase	DHAK_HUMAN	-11
Calcium-binding mitochondrial carrier protein SCaMC-1	SCMC1_HUMAN	-5
Caldesmon CALD1	CALD1_HUMAN	2.9
Calpain-2 catalytic subunit	CAN2_HUMAN	-3.3
Calpastatin	ICAL_HUMAN	4.1
Calponin-1	CNN1_HUMAN	4.7
Calponin-3	CNN3_HUMAN	-10
cAMP-dependent protein kinase type II-alpha regulatory subunit	KAP2_HUMAN	-3.3
Carnitine O-acetyltransferase	CACP_HUMAN	-10
Carnitine O-palmitoyltransferase 2, mitochondrial	CPT2_HUMAN	-3.3
Catechol O-methyltransferase domain-containing protein 1	CMTD1_HUMAN	-10
Cathepsin B	CATB_HUMAN	-2.5
CD59 glycoprotein	CD59_HUMAN	2.2
CDK5 regulatory subunit-associated protein 3	CK5P3_HUMAN	-2.5
Chaperone activity of bc1 complex-like, mitochondrial	ADCK3_HUMAN	2.7
Chromogranin-A	CMGA_HUMAN	-5
Cleavage and polyadenylation specificity factor subunit 5	CPSF5_HUMAN	-2.5
Coatomer subunit epsilon	COPE_HUMAN	-2.5
Coiled-coil domain-containing protein 47	CCD47_HUMAN	-3.3
Coiled-coil-helix-coiled-coil-helix domain-containing protein 3	CHCH3_HUMAN	3.1
Cold-inducible RNA-binding protein	CIRBP_HUMAN	-2
Complement C3	CO3_HUMAN	-2.5
Complement component C9	CO9_HUMAN	-25
Copine-1	CPNE1_HUMAN	-3.3
Core histone macro-H2A.1	H2AY_HUMAN	-2.5
Core histone macro-H2A.2	H2AW_HUMAN	-3.3
Coronin-1B	COR1B_HUMAN	-2.5
Cystathionine beta-synthase	CBS_HUMAN	-2
Cysteinyl-tRNA synthetase, cytoplasmic	SYCC_HUMAN	-2

Supplementary Table 4 continued

Cytoplasmic dynein 1 intermediate chain 2	DC1I2_HUMAN	-5
Cytosol aminopeptidase	AMPL_HUMAN	-3.3
Death-associated protein 1	DAP1_HUMAN	-2.5
Delta-1-pyrroline-5-carboxylate synthase	P5CS_HUMAN	-5
Desmoplakin	DESP_HUMAN	-2.5
Dihydrolipoyllysine-residue acetyltransferase	ODP2_HUMAN	-5
DNA-dependent protein kinase catalytic subunit	PRKDC_HUMAN	-10
Dolichyl-diphosphooligosaccharide--protein glycosyltransferase subunit	STT3A_HUMAN	-2.5
Dolichyl-phosphate beta-glucosyltransferase	ALG5_HUMAN	-2
E3 ubiquitin/ISG15 ligase	TRI25_HUMAN	-2
EH domain-containing protein 2	EHD2_HUMAN	2.9
Electrogenic sodium bicarbonate cotransporter 1	S4A4_HUMAN	-10
EMILIN-1	EMIL1_HUMAN	-12.5
Endothelial differentiation-related factor 1	EDF1_HUMAN	-3.3
Enoyl-CoA delta isomerase 2	ECI2_HUMAN	-5
Enoyl-CoA hydratase domain-containing protein 1	ECHD1_HUMAN	-11
Eosinophil cationic protein	ECP_HUMAN	18
Epithelial cell adhesion molecule	EPCAM_HUMAN	-2.5
ERO1-like protein alpha	ERO1A_HUMAN	-10
ERO1-like protein beta	ERO1B_HUMAN	-2
Eukaryotic translation initiation factor 1	EIF1_HUMAN	-3.3
Eukaryotic translation initiation factor 2 subunit 3	IF2G_HUMAN	-2.5
Eukaryotic translation initiation factor 3 subunit B	EIF3B_HUMAN	-2.5
Eukaryotic translation initiation factor 5	IF5_HUMAN	-5
Extended synaptotagmin-1	ESYT1_HUMAN	-2.5
F-actin-capping protein subunit beta	CAPZB_HUMAN	-2.5
Fatty acid synthase	FAS_HUMAN	3.1
Fatty aldehyde dehydrogenase	AL3A2_HUMAN	-5
Ferritin light chain	FRIL_HUMAN	4.9
Fibrinogen-like protein 1	FGL1_HUMAN	6.6
Filamin-C FLNC	FLNC_HUMAN	5.9
Four and a half LIM domains protein 1	FHL1_HUMAN	-10
Fructose-bisphosphate aldolase B	ALDOB_HUMAN	-2
Fumarate hydratase, mitochondrial	FUMH_HUMAN	-2
Galactokinase	GALK1_HUMAN	2.5
Galectin-2	LEG2_HUMAN	-2
GDP-L-fucose synthase	FCL_HUMAN	-5
General transcription factor II-I	GTF2I_HUMAN	-5
Glucosamine 6-phosphate N-acetyltransferase	GNA1_HUMAN	-2.5
Glucosidase 2 subunit beta	GLU2B_HUMAN	-2
Glutathione peroxidase 1	GPX1_HUMAN	2.6
Glutathione S-transferase Mu 1	GSTM1_HUMAN	4.3
Golgi phosphoprotein 3	GOLP3_HUMAN	-12.5
Golgi resident protein GCP60	GCP60_HUMAN	-5
GTP:AMP phosphotransferase, mitochondrial	KAD3_HUMAN	-2
GTP-binding protein SAR1a	SAR1A_HUMAN	-2.5
GTP-binding protein SAR1b	SAR1B_HUMAN	-2.5
Guanine nucleotide-binding protein G(i) subunit alpha-2	GNAI2_HUMAN	2.2
Guanine nucleotide-binding protein G(I)/G(S)/G(O) subunit gamma-12	GBG12_HUMAN	-2.5

Supplementary Table 4 continued

Guanine nucleotide-binding protein G(I)/G(S)/G(T) subunit beta-1	GBB1_HUMAN	-2.5
Guanine nucleotide-binding protein G(I)/G(S)/G(T) subunit beta-2	GBB2_HUMAN	-2.5
Heme-binding protein 1	HEBP1_HUMAN	-2.5
Heterogeneous nuclear ribonucleoprotein A/B	ROAA_HUMAN	-2
Heterogeneous nuclear ribonucleoprotein A3	ROA3_HUMAN	-2.5
Heterogeneous nuclear ribonucleoprotein F	HNRPF_HUMAN	-2
Heterogeneous nuclear ribonucleoprotein H2	HNRH2_HUMAN	-3.3
High mobility group protein B2	HMGB2_HUMAN	-3.3
Histone H1x	H1X_HUMAN	2.3
HLA class II histocompatibility antigen, DR alpha chain	DRA_HUMAN	-2.5
Hsp90 co-chaperone	CDC37_HUMAN	-12.5
Ig alpha-2 chain C region	IGHA2_HUMAN	6.1
Ig kappa chain V-III region	KV302_HUMAN	-3.3
Importin-5	IPO5_HUMAN	3.4
Inorganic pyrophosphatase 2, mitochondrial	IPYR2_HUMAN	-2
Inosine-5'-monophosphate dehydrogenase 2	IMDH2_HUMAN	-25
Integrin-linked protein kinase	ILK_HUMAN	-2
Inter-alpha-trypsin inhibitor heavy chain H4	ITIH4_HUMAN	-10
Interleukin enhancer-binding factor 2	ILF2_HUMAN	-3.3
Interleukin enhancer-binding factor 3	ILF3_HUMAN	-3.3
Isochorismatase domain-containing protein 2, mitochondrial	ISOC2_HUMAN	-2.5
Isoleucyl-tRNA synthetase, cytoplasmic	SYIC_HUMAN	-2.5
Isoleucyl-tRNA synthetase, mitochondrial	SYIM_HUMAN	-3.3
Junction plakoglobin	PLAK_HUMAN	-2
Lactotransferrin	TRFL_HUMAN	3.6
Lamina-associated polypeptide 2, isoforms beta/gamma	LAP2B_HUMAN	-2
La-related protein 1	LARP1_HUMAN	-2
Leukotriene A-4 hydrolase	LKHA4_HUMAN	-2.5
Lipopolysaccharide-binding protein	LBP_HUMAN	2.8
Liver carboxylesterase 1	EST1_HUMAN	2.1
Lon protease homolog, mitochondrial	LONM_HUMAN	-5
Lupus La protein	LA_HUMAN	-14.3
Lysosome membrane protein 2	SCRB2_HUMAN	-5
Lysozyme C	LYSC_HUMAN	12
Lysyl-tRNA synthetase	SYK_HUMAN	-2.5
Macrophage migration inhibitory factor	MIF_HUMAN	-3.3
Malectin	MLEC_HUMAN	-2.5
Malignant T cell-amplified sequence 1	MCTS1_HUMAN	-10
Mannose-6-phosphate isomerase	MPI_HUMAN	-2.5
Mast cell carboxypeptidase A	CBPA3_HUMAN	2
Matrix metalloproteinase-9	MMP9_HUMAN	5.4
Methyl-CpG-binding protein 2	MECP2_HUMAN	-2.5
Methylcrotonoyl-CoA carboxylase beta chain	MCCB_HUMAN	-11
Methylcrotonoyl-CoA carboxylase subunit alpha	MCCA_HUMAN	-10
Microfibril-associated glycoprotein 4	MFAP4_HUMAN	-11
Microsomal glutathione S-transferase 1	MGST1_HUMAN	-2.5
Microsomal glutathione S-transferase 3	MGST3_HUMAN	-2.5
Mitochondrial 2-oxoglutarate/malate carrier protein	M2OM_HUMAN	-12.5
Mitochondrial antiviral-signaling protein	MAVS_HUMAN	-5

Supplementary Table 4 continued

Myeloblastin	PRTN3_HUMAN	13
Myeloperoxidase	PERM_HUMAN	16
Myosin light chain kinase	MYLK_HUMAN	24
Myosin-10	MYH10_HUMAN	-3.3
Myosin-14	MYH14_HUMAN	-33
Myosin-Ib	MYO1B_HUMAN	-5
Myotrophin	MTPN_HUMAN	-3.3
Myristoylated alanine-rich C-kinase substrate	MARCS_HUMAN	-2.5
N(4)-(beta-N-acetylglucosaminyl)-L-asparaginase	ASPG_HUMAN	-5
Na(+)/H(+) exchange regulatory cofactor NHE-RF1	NHRF1_HUMAN	-2
NAD(P) transhydrogenase	NNTM_HUMAN	-10
NADH dehydrogenase [ubiquinone] 1 alpha subcomplex subunit 5	NDUA5_HUMAN	2
NADH dehydrogenase [ubiquinone] 1 alpha subcomplex subunit 9	NDUA9_HUMAN	-2.5
NADH dehydrogenase [ubiquinone] flavoprotein 1, mitochondrial	NDUV1_HUMAN	-2
NADH dehydrogenase [ubiquinone] iron-sulfur protein 3	NDUS3_HUMAN	2.3
Neutrophil elastase	ELNE_HUMAN	4.8
Neutrophil gelatinase-associated lipocalin	NGAL_HUMAN	12
Nicotinamide phosphoribosyltransferase	NAMPT_HUMAN	-3.3
Nidogen-2	NID2_HUMAN	2.5
Nitrilase homolog 1	NIT1_HUMAN	4.8
Nucleolar protein 58	NOP58_HUMAN	-3.3
Nucleoside diphosphate kinase A	NDKA_HUMAN	-3.3
Olfactomedin-4 OLFM4	OLFM4_HUMAN	-5
Omega-amidase NIT2	NIT2_HUMAN	-3.3
Palladin	PALLD_HUMAN	5.5
PC4 and SFRS1-interacting protein	PSIP1_HUMAN	-5
PDZ and LIM domain protein 3	PDLI3_HUMAN	8.3
Peptidyl-prolyl cis-trans isomerase FKBP2	FKBP2_HUMAN	2.1
Peptidyl-prolyl cis-trans isomerase FKBP4	FKBP4_HUMAN	-2.5
Perilipin-1	PLIN1_HUMAN	9.9
Periostin	POSTN_HUMAN	3.9
Peroxisomal multifunctional enzyme type 2	DHB4_HUMAN	-10
Phosphoenolpyruvate carboxykinase [GTP], mitochondrial	PCKGM_HUMAN	-2
Phosphomannomutase 2	PMM2_HUMAN	-2.5
Plasma protease C1 inhibitor	IC1_HUMAN	-3.3
Platelet glycoprotein 4	CD36_HUMAN	3.3
PRA1 family protein 3	PRAF3_HUMAN	-2.5
Prefoldin subunit 3	PFD3_HUMAN	-5
Pre-mRNA-processing-splicing factor 8	PRP8_HUMAN	-2.5
Prolyl endopeptidase	PPCE_HUMAN	3.4
ProSAAS	PCSK1_HUMAN	2.2
Prostaglandin E synthase 3	TEBP_HUMAN	-2.5
Proteasome subunit alpha type-3	PSA3_HUMAN	-5
Proteasome subunit alpha type-4	PSA4_HUMAN	-2.5
Proteasome subunit beta type-2	PSB2_HUMAN	-3.3
Proteasome subunit beta type-5	PSB5_HUMAN	-5
Proteasome subunit beta type-6	PSB6_HUMAN	-3.3
Proteasome subunit beta type-7	PSB7_HUMAN	-5
Protein AMBP	AMBP_HUMAN	2.3

Protein CDV3 homolog	CDV3_HUMAN	-2.5
Protein dpy-30 homolog	DPY30_HUMAN	-2
Protein FAM98B	FA98B_HUMAN	-2.5
Protein NDRG1	NDRG1_HUMAN	-5
Protein Niban	NIBAN_HUMAN	2.3
Protein NipSnap homolog 1	NIPS1_HUMAN	-2.5
Protein sel-1 homolog 1	SE1L1_HUMAN	-2.5
Protein SET	SET_HUMAN	-2.5
Protein transport protein Sec24C	SC24C_HUMAN	-3.3
Protein transport protein Sec24D	SC24D_HUMAN	-5
Protein transport protein Sec61 subunit beta	SC61B_HUMAN	-2
Protein-L-isoaspartate(D-aspartate) O-methyltransferase	PIMT_HUMAN	2.5
Protein-tyrosine sulfotransferase 2	TPST2_HUMAN	-5
Proteolipid protein 2	PLP2_HUMAN	-2
Prothymosin alpha	PTMA_HUMAN	4
Puromycin-sensitive aminopeptidase	PSA_HUMAN	-2.5
Putative adenosylhomocysteinase 2	SAHH2_HUMAN	-2.5
Putative pre-mRNA-splicing factor ATP-dependent RNA helicase	DHX15_HUMAN	-20
Putative RNA-binding protein 3	RBM3_HUMAN	-2
Pyridoxine-5'-phosphate oxidase	PNPO_HUMAN	-3.3
Ran-specific GTPase-activating protein	RANG_HUMAN	-2
Ras-related protein Rab-10	RAB10_HUMAN	-2.5
Ras-related protein Rab-14	RAB14_HUMAN	-3.3
Ras-related protein Rab-7a	RAB7A_HUMAN	-5
Ras-related protein Ral-A	RALA_HUMAN	4.2
Receptor expression-enhancing protein 5	REEP5_HUMAN	2.2
Regenerating islet-derived protein 3-alpha	REG3A_HUMAN	-3.3
Regucalcin	RGN_HUMAN	-10
Replication protein A 14 kDa subunit	RFA3_HUMAN	10
Reticulocalbin-1	RCN1_HUMAN	-20
Rho GDP-dissociation inhibitor 2	GDIR2_HUMAN	-10
Rho GTPase-activating protein 1	RHG01_HUMAN	-3.3
Ribonuclease UK114	UK114_HUMAN	-5
Ribose-phosphate pyrophosphokinase 1	PRPS1_HUMAN	-14.3
Ribosome maturation protein SBDS	SBDS_HUMAN	2.1
RNA-binding protein 8A	RBM8A_HUMAN	-16.6
SAM domain and HD domain-containing protein 1	SAMH1_HUMAN	-10
Secretogranin-1	SCG1_HUMAN	-5
Selenocysteine lyase	SCLY_HUMAN	2
Septin-11	SEP11_HUMAN	-10
Septin-2	SEPT2_HUMAN	-3.3
Serine hydroxymethyl transferase, mitochondrial	GLYM_HUMAN	-2.5
Serine/arginine-rich splicing factor 1	SRSF1_HUMAN	-5
Serine/arginine-rich splicing factor 2	SRSF2_HUMAN	-2.5
Serine/arginine-rich splicing factor 9	SRSF9_HUMAN	-2.5
Serine/threonine-protein phosphatase 2A catalytic subunit alpha isoform	PP2AA_HUMAN	-2
Serum amyloid P-component	SAMP_HUMAN	-10
S-formylglutathione hydrolase	ESTD_HUMAN	-2.5
Short/branched chain specific acyl-CoA dehydrogenase	ACDSB_HUMAN	-5

Supplementary Table 4 continued

Sorbin and SH3 domain-containing protein 1	SRBS1_HUMAN	10
Spermidine synthase	SPEE_HUMAN	-33
Splicing factor 3B subunit 1	SF3B1_HUMAN	-2.5
Splicing factor U2AF 35 kDa subunit	U2AF1_HUMAN	-3.3
Stress-induced-phosphoprotein 1	STIP1_HUMAN	-5
Succinyl-CoA ligase [GDP-forming] subunit alpha, mitochondrial	SUCA_HUMAN	-3.3
SUN domain-containing protein 2	SUN2_HUMAN	-2.5
Thioredoxin	THIO_HUMAN	-2
Thiosulfate sulfurtransferase/rhodanese-like domain-containing protein 1	TSTD1_HUMAN	-2.5
Translocon-associated protein subunit beta	SSRB_HUMAN	-3.3
Transmembrane emp24 domain-containing protein 2	TMED2_HUMAN	-2.5
Trifunctional purine biosynthetic protein adenosine-3	PUR2_HUMAN	-5
tRNA-splicing ligase RtcB homolog.	RTCB_HUMAN	-11
Tropomyosin alpha-1 chain	TPM1_HUMAN	2.1
Tumor-associated calcium signal transducer 2	TACD2_HUMAN	-20
Tyrosyl-tRNA synthetase, cytoplasmic	SYYC_HUMAN	-2
U2 small nuclear ribonucleoprotein A	RU2A_HUMAN	-10
Ubiquitin carboxyl-terminal hydrolase 14	UBP14_HUMAN	-20
Ubiquitin carboxyl-terminal hydrolase 5	UBP5_HUMAN	-2.5
Ubiquitin-conjugating enzyme E2 L3	UB2L3_HUMAN	-3.3
Ubiquitin-conjugating enzyme E2 variant 2	UB2V2_HUMAN	-2.5
Ubiquitin-like modifier-activating enzyme 5	UBA5_HUMAN	-2
Utrophin	UTRO_HUMAN	-2.5
Vacuolar protein sorting-associated protein 35	VPS35_HUMAN	-3.3
Vesicle-associated membrane protein 2	VAMP2_HUMAN	2.9
Vesicle-associated membrane protein 8	VAMP8_HUMAN	6.4
Voltage-dependent anion-selective channel protein 2	VDAC2_HUMAN	-2.5
V-type proton ATPase catalytic subunit A	VATA_HUMAN	-2.5
Zyxin	ZYX_HUMAN	2.8
