# Supplementary material for: Proteomic Analysis of Disease Stratified Human Pancreas Tissue Indicates Unique Signature of Type 1 Diabetes
Source: PLoS One. 2015 Aug 24;10(8):e0135663. doi: 10.1371/journal.pone.0135663 (PMC4547762; doi:10.1371/journal.pone.0135663)
Supplement: S5 Table — (PDF) [file pone.0135663.s015.pdf]

S5 Table. List of differentially regulated proteins between no disease (ND) and type 1 diabetes (T1D) cases. Only proteins with a change of 2 fold or more are included in this list. P*<0.05.

**Protein Description**	**Accession Number**	**Fold Change* T1D vs ND**
14 kDa phosphohistidine phosphatase	PHP14_HUMAN	-2.5
26S protease regulatory subunit 6B	PRS6B_HUMAN	7.3
26S proteasome non ATPase regulatory subunit 5	PSMD5_HUMAN	2.1
40S ribosomal protein S26	RS26_HUMAN	2.5
40S ribosomal protein S30	RS30_HUMAN	-3.3
60 kDa SSA/Ro ribonucleoprotein	RO60_HUMAN	6.4
60S ribosomal protein L29	RL29_HUMAN	4.2
60S ribosomal protein L34	RL34_HUMAN	-2.5
Acid ceramidase	ASAH1_HUMAN	2.7
Acid sphingomyelinase like phosphodiesterase 3b	ASM3B_HUMAN	4
Acidic leucine rich nuclear phosphoprotein 32	AN32B_HUMAN	13
Actin related protein 2	ARP2_HUMAN	-2
Acylcoenzyme A thioesterase 1	ACOT1_HUMAN	2.7
Adipocyte plasma membrane associated protein	APMAP_HUMAN	4
ADP ribosylation factor 4	ARF4_HUMAN	-2.5
ADP ribosylation factor 6	ARF6_HUMAN	2.8
Alcohol dehydrogenase 1C	ADH1G_HUMAN	-16.6
Aldehyde dehydrogenase family 1 member L1	AL1L1_HUMAN	-10
Aldose reductase	ALDR_HUMAN	-2.5
Alpha1Bglycoprotein	A1BG_HUMAN	5.2
Alpha adducin	ADDA_HUMAN	2.6

Aminopeptidase N	AMPN_HUMAN	-3.3
Angiotensinogen	ANGT_HUMAN	-14
Anterior gradient protein 2 homolog	AGR2_HUMAN	-12.5
Antithrombin III	ANT3_HUMAN	3.1
AP1 complex subunit beta1	AP1B1_HUMAN	-2
AP2 complex subunit alpha2	AP2A2_HUMAN	2.1
AP2 complex subunit mu	AP2M1_HUMAN	-3.3
Apolipoprotein AII	APOA2_HUMAN	4.5
Apolipoprotein CIII	APOC3_HUMAN	5.2
Apolipoprotein E	APOE_HUMAN	3.9
Apoptosis inhibitor 5	API5_HUMAN	-2
Aquaporin1	AQP1_HUMAN	2.8
Arylsulfatase A	ARSA_HUMAN	-10
Arylsulfatase E	ARSE_HUMAN	7.2
Asporin	ASPN_HUMAN	5.4
ATP synthase subunit gamma, mitochondrial	ATPG_HUMAN	-2
ATP dependent RNA helicase DDX1	DDX1_HUMAN	-2
Basal cell adhesion molecule	BCAM_HUMAN	9.7
B cell receptor associated protein 31	BAP31_HUMAN	-2
Bifunctional ATP dependent dihydroxyacetone kinase	DHAK_HUMAN	-5
Biglycan	PGS1_HUMAN	2.4
BTB/POZ domain containing protein KCTD12	KCD12_HUMAN	2.8
Calpastatin	ICAL_HUMAN	11
Calponin1	CNN1_HUMAN	3
Carbonic anhydrase 1	CAH1_HUMAN	-3.3

Supplementary Table 5 continued

Carboxypeptidase E	CBPE_HUMAN	-10
Carnitine O acetyltransferase	CACP_HUMAN	-5
Carnitine O palmitoyltransferase 2, mitochondrial	CPT2_HUMAN	-3.3
Cathepsin B	CATB_HUMAN	-2.5
Caveolin1	CAV1_HUMAN	4.7
CD59	CD59_HUMAN	3.9
CD9	CD9_HUMAN	2
Chaperone activity of bc1 complex like, mitochondrial	ADCK3_HUMAN	3.1
Cleavage and polyadenylation specificity factor subunit 5	CPSF5_HUMAN	8.2
Collagen alpha3(IV) chain	CO4A3_HUMAN	8.6
Complement C5	CO5_HUMAN	6.3
Complement component C7	CO7_HUMAN	5.6
Complement component C8 alpha chain	CO8A_HUMAN	9.4
Complement component C9	CO9_HUMAN	4.3
Copine1	CPNE1_HUMAN	2.6
Cystathionine beta synthase	CBS_HUMAN	-3.3
Cystatin B	CYTB_HUMAN	3
Cysteine rich protein 2	CRIP2_HUMAN	4.1
Cytochrome c1, heme protein, mitochondrial	CY1_HUMAN	-2
Cytoplasmic dynein 1 intermediate chain 2	DC1I2_HUMAN	-5
Delta1pyrroline5carboxylate synthase	P5CS_HUMAN	-2.5
Desmin	DESM_HUMAN	-2.5
Destrin	DEST_HUMAN	-2.5
Dihydrolipoyllysineresidue acetyltransferase	ODP2_HUMAN	-3.3
DNA(apurinic or apyrimidinic site) lyase	APEX1_HUMAN	2.6

Supplementary Table 5 continued

DNA dependent protein kinase catalytic subunit	PRKDC_HUMAN	-2
DnaJ homolog subfamily C member 3	DNJC3_HUMAN	-10
Dolichyldiphosphooligosaccharideprotein glycosyltransferase	STT3A_HUMAN	-2
Ectonucleotide pyrophosphatase/phosphodiesterase member 1	ENPP1_HUMAN	2.1
EH domain containing protein 2	EHD2_HUMAN	4.2
Electrogenic sodium bicarbonate cotransporter 1	S4A4_HUMAN	-3.3
EnoylCoA delta isomerase 2, mitochondrial	ECI2_HUMAN	2.5
EnoylCoA hydratase domain containing protein 1	ECHD1_HUMAN	-2.5
ERO1like protein beta	ERO1B_HUMAN	-3.3
ES1 protein homolog, mitochondrial	ES1_HUMAN	3.3
Ester hydrolase C11orf54	CK054_HUMAN	2.8
Eukaryotic initiation factor 4AII	IF4A2_HUMAN	2.4
Eukaryotic translation initiation factor 3 subunit H	EIF3H_HUMAN	5
Eukaryotic translation initiation factor 3 subunit J	EIF3J_HUMAN	2.5
Eukaryotic translation initiation factor 4B	IF4B_HUMAN	5.3
Extended synaptotagmin1	ESYT1_HUMAN	-2.5
Extracellular matrix protein FRAS1	FRAS1_HUMAN	16
Far upstream element binding protein 1	FUBP1_HUMAN	2
Far upstream element binding protein 2	FUBP2_HUMAN	2.3
Fatty aldehyde dehydrogenase	AL3A2_HUMAN	-3.3
Fermitin family homolog 2	FERM2_HUMAN	6.1
Ferritin light chain	FRIL_HUMAN	19
Fibrinogen like protein 1	FGL1_HUMAN	10
Fibulin1	FBLN1_HUMAN	3.3
Filamin C	FLNC_HUMAN	3.7

Four and a half LIM domains protein 1	FHL1_HUMAN	3.9
Fructose bisphosphate aldolase B	ALDOB_HUMAN	3.9
Fructose bisphosphate aldolase C	ALDOC_HUMAN	2.5
Galactokinase	GALK1_HUMAN	3.5
Galectin3	LEG3_HUMAN	-3.3
GDP mannose 4,6 dehydratase	GMDS_HUMAN	3.2
General transcription factor III	GTF2I_HUMAN	-3.3
Glucosamine 6phosphate N acetyltransferase	GNA1_HUMAN	-3.3
Glutaminase kidney isoform, mitochondrial	GLSK_HUMAN	5.8
Glutathione peroxidase 1	GPX1_HUMAN	5.1
Golgi phosphoprotein 3	GOLP3_HUMAN	-16.6
Golgi resident protein GCP60	GCP60_HUMAN	-12.5
Guanine nucleotide binding protein G(i) subunit alpha2	GNAI2_HUMAN	2.4
H/ACA ribonucleoprotein complex subunit 4	DKC1_HUMAN	6.7
Hematopoietic lineage cell specific protein	HCLS1_HUMAN	7.1
Hemebinding protein 2	HEBP2_HUMAN	2
Hemoglobin subunit delta	HBD_HUMAN	2.9
Heterogeneous nuclear ribonucleoprotein A/B	ROAA_HUMAN	5.2
Heterogeneous nuclear ribonucleoprotein R	HNRPR_HUMAN	2
Histone H1x	H1X_HUMAN	2.2
HLA class I histocompatibility antigen, Cw12 alpha chain	1C12_HUMAN	2.1
HLA class II histocompatibility antigen, DR alpha chain	DRA_HUMAN	6
HydroxymethylglutarylCoA synthase, mitochondrial	HMCS2_HUMAN	3.4
Hypoxanthineguanine phosphoribosyltransferase	HPRT_HUMAN	2.4
Ig alpha2 chain C region	IGHA2_HUMAN	15

Supplementary Table 5 continued

Ig gamma2 chain C region	IGHG2_HUMAN	2.6
Ig gamma4 chain C region	IGHG4_HUMAN	2.8
Inosine5'monophosphate dehydrogenase 2	IMDH2_HUMAN	-2.5
Inositol monophosphatase 2	IMPA2_HUMAN	-3.3
Inositol3phosphate synthase 1	INO1_HUMAN	2.1
Insulin	INS_HUMAN	-5
Interalphatrypsin inhibitor heavy chain H4	ITIH4_HUMAN	-10
Isochorismatase domain containing protein 2, mitochondrial	ISOC2_HUMAN	-2.5
Isocitrate dehydrogenase [NAD] subunit alpha, mitochondrial	IDH3A_HUMAN	-3.3
IsoleucyltRNA synthetase, mitochondrial	SYIM_HUMAN	-2
Junctional adhesion molecule A	JAM1_HUMAN	4.9
Keratin, type I cytoskeletal 10	K1C10_HUMAN	-2
Kinectin	KTN1_HUMAN	11
Lactotransferrin	TRFL_HUMAN	2.7
LaminB2	LMNB2_HUMAN	2.5
La related protein 1	LARP1_HUMAN	-2.5
Lipase maturation factor 2	LMF2_HUMAN	10
Lipoma preferred partner	LPP_HUMAN	3.9
Lon protease homolog, mitochondrial	LONM_HUMAN	-2.5
Lupus La protein	LA_HUMAN	-2
Lxylulose reductase	DCXR_HUMAN	-3.3
Lysosome membrane protein 2	SCRB2_HUMAN	-11
Lysozyme C	LYSC_HUMAN	15
MACRO domain containing protein 1	MACD1_HUMAN	2.3
Malectin	MLEC_HUMAN	-2.5

Malignant T cell amplified sequence 1	MCTS1_HUMAN	-10
Mannose6phosphate isomerase	MPI_HUMAN	2.6
Mast cell carboxypeptidase A	CBPA3_HUMAN	2.4
Metallothionein1E	MT1E_HUMAN	3.2
Methionyl tRNA synthetase, cytoplasmic	SYMC_HUMAN	-2.5
MethylCpGbinding protein 2	MECP2_HUMAN	2
MethylcrotonoylCoA carboxylase subunit alpha, mitochondrial	MCCA_HUMAN	-2.5
Microfibril associated glycoprotein 4	MFAP4_HUMAN	-5
Microsomal glutathione S transferase 1	MGST1_HUMAN	-5
Microsomal glutathione S transferase 3	MGST3_HUMAN	-2.5
Mitochondrial 2oxoglutarate/malate carrier protein	M2OM_HUMAN	-3.3
Mitochondrial antiviral signaling protein	MAVS_HUMAN	-2
Mitochondrial import receptor subunit TOM70	TOM70_HUMAN	-2
Mitochondrial inner membrane protein	IMMT_HUMAN	-5
Myeloblastin	PRTN3_HUMAN	8.4
Myeloperoxidase	PERM_HUMAN	14
Myosin14	MYH14_HUMAN	-3.3
Myosin Ib	MYO1B_HUMAN	-2.5
N(G),N(G)dimethylarginine dimethylaminohydrolase 2	DDAH2_HUMAN	4.8
NAD(P) transhydrogenase, mitochondrial	NNTM_HUMAN	-2
NADH dehydrogenase [ubiquinone] 1 alpha subcomplex subunit 4	NDUA4_HUMAN	-2.5
NADH dehydrogenase [ubiquinone] flavoprotein 1, mitochondrial	NDUV1_HUMAN	-5
NADH cytochrome b5 reductase 3	NB5R3_HUMAN	2.2
Neutrophil elastase	ELNE_HUMAN	-2
Nidogen2	NID2_HUMAN	3.9

Supplementary Table 5 continued

Non POU domain containing octamer binding protein	NONO_HUMAN	4
Nucleolar protein 58	NOP58_HUMAN	2.2
Obglike ATPase 1	OLA1_HUMAN	2.8
Olfactomedin4	OLFM4_HUMAN	4.7
Pancreatic prohormone	PAHO_HUMAN	-3.3
PDZ and LIM domain protein 1	PDLI1_HUMAN	3.4
PDZ and LIM domain protein 3	PDLI3_HUMAN	7
Peptidylprolyl cistrans isomerase F	PPIF_HUMAN	-14
Peptidylprolyl cistrans isomerase FKBP4	FKBP4_HUMAN	-3.3
Periostin	POSTN_HUMAN	11
Phenylalanyl tRNA synthetase alpha chain	SYFA_HUMAN	-2.5
Phosphoserine aminotransferase	SERC_HUMAN	-2
Probable ATP dependent RNA helicase DDX5	DDX5_HUMAN	3.8
Probable serine carboxypeptidase CPVL	CPVL_HUMAN	-14
Proline synthase cotranscribed bacterial homolog protein	PROSC_HUMAN	2.7
Prolyl endopeptidase	PPCE_HUMAN	3.7
PropionylCoA carboxylase beta chain, mitochondrial	PCCB_HUMAN	-14
ProSAAS	PCSK1_HUMAN	2.8
Proteasome subunit alpha type1	PSA1_HUMAN	2.3
Proteasome subunit alpha type3	PSA3_HUMAN	-2
Proteasome subunit alpha type4	PSA4_HUMAN	-2.5
Proteasome subunit beta type1	PSB1_HUMAN	-2.5
Proteasome subunit beta type5	PSB5_HUMAN	-5
Proteasome subunit beta type6	PSB6_HUMAN	-10
Proteasome subunit beta type7	PSB7_HUMAN	-2

Protein AMBP	AMBP_HUMAN	4.1
Protein FAM98B	FA98B_HUMAN	2.9
Protein NipSnap homolog 2	NIPS2_HUMAN	5.1
Protein sel1 homolog 1	SE1L1_HUMAN	-2.5
Prothymosin alpha	PTMA_HUMAN	-2.5
Putative adenosylhomocysteinase 2	SAHH2_HUMAN	-20
Putative tropomyosin alpha3 chainlike protein	TPM3L_HUMAN	2.2
Ras related protein Rab14	RAB14_HUMAN	-2.5
Ras related protein RalA	RALA_HUMAN	11
Receptor expression enhancing protein 5	REEP5_HUMAN	2.4
Regenerating islet derived protein 3gamma	REG3G_HUMAN	2
Replication protein A 14 kDa subunit	RFA3_HUMAN	4.6
Rho GTPase activating protein 1	RHG01_HUMAN	-2.5
Ribonuclease UK114	UK114_HUMAN	-5
Ribosephosphate pyrophosphokinase 1	PRPS1_HUMAN	-10
Ribosome maturation protein SBDS	SBDS_HUMAN	-2.5
RNA binding protein 39	RBM39_HUMAN	3.3
RNA binding protein 8A	RBM8A_HUMAN	-16.6
RNA binding protein FUS	FUS_HUMAN	2.8
RNA binding protein with serine rich domain 1	RNPS1_HUMAN	2.8
SAP domain containing ribonucleoprotein	SARNP_HUMAN	8.6
Secretogranin1	SCG1_HUMAN	-11
Selenocysteine lyase	SCLY_HUMAN	9.1
Serine/arginine rich splicing factor 10	SRS10_HUMAN	2.8
Serum amyloid P component	SAMP_HUMAN	3.3

Supplementary Table 5 continued

Short/branched chain specific acyl CoA dehydrogenase	ACDSB_HUMAN	-3.3
Signal recognition particle 54 kDa protein	SRP54_HUMAN	-5
Splicing factor 3B subunit 1	SF3B1_HUMAN	2.4
Stathmin	STMN1_HUMAN	6.7
Sulfide:quinone oxidoreductase, mitochondrial	SQRD_HUMAN	-3.3
T complex protein 1 subunit alpha	TCPA_HUMAN	-2.5
T complex protein 1 subunit epsilon	TCPE_HUMAN	-2.5
T complex protein 1 subunit eta	TCPH_HUMAN	-2
Thioredoxin domain containing protein 17	TXD17_HUMAN	7
Thymidine phosphorylase	TYPH_HUMAN	4.1
Transcription factor BTF3 homolog 4	BT3L4_HUMAN	-3.3
Transmembrane 9 superfamily member 2	TM9S2_HUMAN	-2.5
Transmembrane emp24 domain containing protein 2	TMED2_HUMAN	-2.5
Transmembrane protein 205	TM205_HUMAN	-10
Tubulin specific chaperone A	TBCA_HUMAN	2.9
U5 small nuclear ribonucleoprotein 200 kDa helicase	U520_HUMAN	2.6
Ubiquitin conjugating enzyme E2 variant 2	UB2V2_HUMAN	2
UPF0556 protein C19orf10	CS010_HUMAN	-2.5
UPF0577 protein KIAA1324	K1324_HUMAN	-2.5
Vasodilator stimulated phosphoprotein	VASP_HUMAN	2.6
Vitronectin	VTNC_HUMAN	3.3
Zyxin	ZYX_HUMAN	3.5
