# Supplementary material for: Proteomic Analysis of Disease Stratified Human Pancreas Tissue Indicates Unique Signature of Type 1 Diabetes
Source: PLoS One. 2015 Aug 24;10(8):e0135663. doi: 10.1371/journal.pone.0135663 (PMC4547762; doi:10.1371/journal.pone.0135663)
Supplement: S6 Table — (PDF) [file pone.0135663.s016.pdf]

S6 Table. List of differentially regulated proteins between no disease (ND) and type 2 diabetes (T2D) cases. Only proteins with a change of 2 fold or more are included in this list. P*<0.05.

Protein Description	Accession Number	Fold Change* T2D vs ND
14 kDa phosphohistidine phosphatase	PHP14_HUMAN	-20
14-3-3 protein eta	1433F_HUMAN	-3.3
26S protease regulatory subunit	PRS6A_HUMAN	-2.5
26S protease regulatory subunit 6	PRS6B_HUMAN	-2.6
26S protease regulatory subunit 8	PRS8_HUMAN	-3.3
26S proteasome non-ATPase regulatory subunit 3	PSMD3_HUMAN	-10
26S proteasome non-ATPase regulatory subunit 7	PSD7_HUMAN	-10
2-amino-3-ketobutyrate coenzyme A ligase	KBL_HUMAN	-3.3
2-oxoisovalerate dehydrogenase subunit alpha	ODBA_HUMAN	-11
3-hydroxyisobutyryl-CoA hydrolase	HIBCH_HUMAN	-3.3
40S ribosomal protein	RS30_HUMAN	-5
60 kDa SS-A/Ro ribonucleoprotein	RO60_HUMAN	5.8
60S ribosomal protein L29	RL29_HUMAN	3
60S ribosomal protein L37a	RL37A_HUMAN	-5
60S ribosomal protein L3-like	RL3L_HUMAN	15
Acid ceramidase	ASAH1_HUMAN	-10
Acid sphingomyelinase-like phosphodiesterase 3b	ASM3B_HUMAN	8.6
Acidic leucine-rich nuclear phosphoprotein 32	AN32B_HUMAN	26
Actin-related protein 2	ARP2_HUMAN	-3.3
Actin-related protein 2/3 complex subunit	ARC1B_HUMAN	-3.3
Acyl-CoA-binding protein	ACBP_HUMAN	-3.3
Acyl-protein thioesterase 1	LYPA1_HUMAN	-25
Acyl-protein thioesterase 2	LYPA2_HUMAN	-5
ADP/ATP translocase 1	ADT1_HUMAN	-10
Aldehyde dehydrogenase X	AL1B1_HUMAN	-3.3
Aldo-keto reductase family 1	AK1C3_HUMAN	2.5
Aldose reductase	ALDR_HUMAN	-50
Alpha-1,2-mannosyltransferase	ALG9_HUMAN	-3.3
Alpha-aminoadipic semialdehyde dehydrogenase	AL7A1_HUMAN	-3.3
Alpha-crystallin B chain	CRYAB_HUMAN	-2.5
Anterior gradient protein 2 homolog	AGR2_HUMAN	-5
Antithrombin-III	ANT3_HUMAN	12
AP-1 complex subunit beta	AP1B1_HUMAN	-5
AP-2 complex subunit mu	AP2M1_HUMAN	-33
Apolipoprotein A-II	APOA2_HUMAN	4
Apolipoprotein C-III	APOC3_HUMAN	4.8
Apolipoprotein E	APOE_HUMAN	7.7
Aspartyl aminopeptidase	DNPEP_HUMAN	-10
Asporin	ASPN_HUMAN	5

Supplementary Table 6 continued.

Astrocytic phosphoprotein PEA-15	PEA15_HUMAN	-10
Atlastin-3	ATLA3_HUMAN	-2.5
ATP synthase subunit f, mitochondrial	ATPK_HUMAN	-2.5
ATP-binding cassette sub-family E	ABCE1_HUMAN	-2.5
ATP-dependent RNA helicase	DDX1_HUMAN	-2.5
B-cell receptor-associated protein 31	BAP31_HUMAN	-2.5
Beta-2-glycoprotein 1	APOH_HUMAN	3.8
Bifunctional ATP-dependent dihydroxyacetone kinase	DHAK_HUMAN	2.3
BTB/POZ domain-containing protein	KCD12_HUMAN	2.5
Calcium-binding mitochondrial carrier protein S	SCMC1_HUMAN	-5
Calpain-2 catalytic subunit	CAN2_HUMAN	2.9
Calponin-1	CNN1_HUMAN	-5
Calponin-3	CNN3_HUMAN	-10
Carbonic anhydrase 1	CAH1_HUMAN	-14.2
Carboxypeptidase E	CBPE_HUMAN	2.2
Carnitine O-palmitoyltransferase 2	CPT2_HUMAN	-2.5
Catalase	CATA_HUMAN	-2
Caveolin-1	CAV1_HUMAN	4.5
CD59	CD59_HUMAN	5.3
CD9	CD9_HUMAN	2.1
Cellular nucleic acid-binding protein	CNBP_HUMAN	2.1
Coiled-coil domain-containing protein 47	CCD47_HUMAN	-5
Cold-inducible RNA-binding protein	CIRBP_HUMAN	-2.5
Collagen alpha-3(IV) chain	CO4A3_HUMAN	5.2
Complement component C9	CO9_HUMAN	2.1
Complement component C7	CO7_HUMAN	-2.5
Copine-1	CPNE1_HUMAN	-3.3
Cysteine-rich protein 2	CRIP2_HUMAN	3.2
Cysteinyl-tRNA synthetase	SYCC_HUMAN	-12.5
Cytochrome b5	CYB5_HUMAN	-3.3
Cytochrome b-c1 complex	UCRI_HUMAN	4.1
Cytochrome c oxidase subunit	COX2_HUMAN	2.4
Cytochrome c oxidase subunit 5A	COX5A_HUMAN	-2
Cytochrome c oxidase subunit 7A2	CX7A2_HUMAN	-2.5
Cytochrome c1, heme protein	CY1_HUMAN	-2.5
Delta-1-pyrroline-5-carboxylate synthase	P5CS_HUMAN	-2.5
Developmentally-regulated GTP-binding protein	DRG1_HUMAN	32
Dihydrolipoyllysine-residue acetyltransferase	ODP2_HUMAN	-25
Dolichyl-diphosphooligosaccharide-protein glycosyltransferase	STT3A_HUMAN	-3.3
Dolichyl-phosphate beta-glucosyltransferase	ALG5_HUMAN	-50
Dystroglycan	DAG1_HUMAN	21
E3 ubiquitin/ISG15 ligase	TRI25_HUMAN	-2
Ectonucleotide pyrophosphatase/phosphodiesterase 1	ENPP1_HUMAN	-2
EH domain-containing protein 2	EHD2_HUMAN	2.7
Electrogenic sodium bicarbonate cotransporter	S4A4_HUMAN	-2.5
Enoyl-CoA delta isomerase 1	ECI1_HUMAN	-5
Epididymal secretory protein E	NPC2_HUMAN	-16.6
Erythrocyte band 7 integral membrane protein	STOM_HUMAN	-3.3
Ester hydrolase C11orf54	CK054_HUMAN	2

Supplementary Table 6 continued.

Estradiol 17-beta-dehydrogenase 11	DHB11_HUMAN	2.7
Eukaryotic initiation factor 4A-II	IF4A2_HUMAN	-2
Eukaryotic translation initiation factor 2 subunit 3	IF2G_HUMAN	-2
Eukaryotic translation initiation factor 3 subunit H	EIF3H_HUMAN	5.2
Eukaryotic translation initiation factor 4 gamma 1	IF4G1_HUMAN	-2.5
Eukaryotic translation initiation factor 4B	IF4B_HUMAN	2.4
Eukaryotic translation initiation factor 5	IF5_HUMAN	-2.5
Eukaryotic translation initiation factor 6	IF6_HUMAN	3.3
Extracellular matrix protein FRAS1	FRAS1_HUMAN	7.4
F-actin-capping protein subunit beta	CAPZB_HUMAN	-3.3
Far upstream element-binding protein	FUBP1_HUMAN	2.3
Far upstream element-binding protein	FUBP2_HUMAN	2.1
Fatty acid synthase	FAS_HUMAN	2.4
Fatty acid-binding protein, adipocyte	FABP4_HUMAN	4
Fermitin family homolog 2	FERM2_HUMAN	4.9
Ferritin light chain	FRIL_HUMAN	7.4
Fibronectin	FINC_HUMAN	-2.5
Fibulin-1	FBLN1_HUMAN	2.5
Four and a half LIM domains protein	FHL1_HUMAN	2.4
Fructose-bisphosphate aldolase	ALDOC_HUMAN	2.1
Fructose-bisphosphate aldolase B	ALDOB_HUMAN	4.4
Galactokinase	GALK1_HUMAN	2.6
Galectin-3	LEG3_HUMAN	-5
Gamma-glutamyltransferase 5	GGT5_HUMAN	2
GDP-L-fucose synthase	FCL_HUMAN	-2.5
Glutaminase kidney isoform, mitochondrial	GLSK_HUMAN	4.4
Glutathione S-transferase kappa 1	GSTK1_HUMAN	-2.5
Glutathione S-transferase Mu 2	GSTM2_HUMAN	2.6
Glutathione S-transferase theta-2	GSTT2_HUMAN	-3.3
Golgi phosphoprotein 3	GOLP3_HUMAN	-11
Golgi resident protein GCP60	GCP60_HUMAN	3.2
GTP-binding protein SAR1b	SAR1B_HUMAN	-2
Guanine nucleotide-binding protein G(i) subunit alpha	GNAI2_HUMAN	2.5
Hemoglobin subunit delta	HBD_HUMAN	3.1
Heterogeneous nuclear ribonucleoprotein A	ROAA_HUMAN	2.5
Heterogeneous nuclear ribonucleoprotein H2	HNRH2_HUMAN	-50
Heterogeneous nuclear ribonucleoprotein U-like protein 1	HNRL1_HUMAN	-2.5
High mobility group protein	HMGB2_HUMAN	-25
Histidine triad nucleotide-binding protein 2	HINT2_HUMAN	2.4
Histone H1x	H1X_HUMAN	3.5
HLA class I histocompatibility antigen	1C12_HUMAN	-2
HLA class I histocompatibility antigen, A-69	1A69_HUMAN	-10
HLA class II histocompatibility antigen, DR alpha chain	DRA_HUMAN	-2.5
Hsp90 co-chaperone	CDC37_HUMAN	-20
Hydroxymethylglutaryl-CoA synthase	HMCS2_HUMAN	2.4
Ig gamma-2 chain C region	IGHG2_HUMAN	2.6
Ig gamma-4 chain C region	IGHG4_HUMAN	-20
Ig heavy chain V-III region BRO	HV305_HUMAN	2.3
Ig mu chain C region	IGHM_HUMAN	-16.6

Supplementary Table 6 continued.

Inorganic pyrophosphatase 2	IPYR2_HUMAN	-3.3
Inorganic pyrophosphatasePPA1	IPYR_HUMAN	-2
Inosine-5'-monophosphate dehydrogenase 2	IMDH2_HUMAN	-2.5
Insulin	INS_HUMAN	-5
Integrin beta-1	ITB1_HUMAN	-3.3
Inter-alpha-trypsin inhibitor heavy chain H	ITIH4_HUMAN	-10
Interleukin enhancer-binding factor 2ILF2	ILF2_HUMAN	-2
Isocitrate dehydrogenase [NAD] subunit alpha	IDH3A_HUMAN	-5
Isoleucyl-tRNA synthetase	SYIC_HUMAN	-2.5
Junctional adhesion molecule	JAM1_HUMAN	14
Keratin, type I cytoskeletal 10	K1C10_HUMAN	-5
Keratin, type I cytoskeletal 9	K1C9_HUMAN	-3.3
Kinesin-1 heavy chain	KINH_HUMAN	-2.5
Lactotransferrin	TRFL_HUMAN	2.1
Lamin-B2	LMNB2_HUMAN	2.4
Laminin subunit alpha-5	LAMA5_HUMAN	2.1
La-related protein 1	LARP1_HUMAN	-20
Lipase maturation factor 2	LMF2_HUMAN	5.8
Lipoamide acyltransferase	ODB2_HUMAN	-14.2
Liver carboxylesterase 1	EST1_HUMAN	3.7
Lon protease homolog	LONM_HUMAN	-25
Long-chain-fatty-acid--CoA ligase 1	ACSL1_HUMAN	-5
Lupus La protein	LA_HUMAN	-2.5
L-xylulose reductase	DCXR_HUMAN	-2
Lysosome membrane protein 2	SCRB2_HUMAN	-50
Lysozyme	LYSC_HUMAN	6.1
MACRO domain-containing protein 1	MACD1_HUMAN	2
Macrophage-capping protein	CAPG_HUMAN	-10
Malectin	MLEC_HUMAN	-3.3
Malignant T cell-amplified sequence 1	MCTS1_HUMAN	-10
Mannose-6-phosphate isomerase	MPI_HUMAN	-2
Metallothionein-1	MT1E_HUMAN	5.6
Microfibril-associated glycoprotein 4	MFAP4_HUMAN	-10
Microsomal glutathione S-transferase 3	MGST3_HUMAN	-2.5
Mitochondrial 2-oxoglutarate/malate carrier protein	M2OM_HUMAN	-3.3
Mitochondrial antiviral-signaling protein	MAVS_HUMAN	-5
Mitochondrial fission 1	FIS1_HUMAN	2.5
Mitochondrial inner membrane protein	IMMT_HUMAN	-2
Moesin	MOES_HUMAN	-2
Myeloperoxidase	PERM_HUMAN	7.7
Myosin-14	MYH14_HUMAN	-11
N(4)-(beta-N-acetylglucosaminyl)-L-asparaginase	ASPG_HUMAN	-20
N-acetyl-D-glucosamine kinase	NAGK_HUMAN	2.2
NAD(P) transhydrogenase	NNTM_HUMAN	2.8
NADH dehydrogenase [ubiquinone] 1 alpha subunit 10,	NDUAA_HUMAN	2
NADH dehydrogenase [ubiquinone] 1 alpha subunit 9	NDUA9_HUMAN	-5
NADH dehydrogenase [ubiquinone] 1 beta subunit 8	NDUB8_HUMAN	-10
NADH dehydrogenase [ubiquinone] flavoprotein 1	NDUV1_HUMAN	-5
NEDD8	NEDD8_HUMAN	-3.3

Supplementary Table 6 continued.

Nicotinamide phosphoribosyltransferaseNAMPT	NAMPT_HUMAN	-2
Nidogen-2	NID2_HUMAN	3.1
Nitrilase homolog 1	NIT1_HUMAN	-3.3
Non-POU domain-containing octamer-binding protein	NONO_HUMAN	5.2
Nuclear transport factor 2	NTF2_HUMAN	-2
Nucleolar protein 56	NOP56_HUMAN	2.3
Olfactomedin-4	OLFM4_HUMAN	-5
PCTP-like protein	PCTL_HUMAN	2
PDZ and LIM domain protein 1	PDLI1_HUMAN	-10
PDZ domain-containing protein	GIPC2_HUMAN	2.1
Peptidyl-prolyl cis-trans isomerase	FKBP4_HUMAN	-2.5
Peptidyl-prolyl cis-trans isomerase F	PPIF_HUMAN	-12.5
Perilipin-1	PLIN1_HUMAN	21
Phenylalanyl-tRNA synthetase alpha chain	SYFA_HUMAN	-5
Phosphate carrier protein	MPCP_HUMAN	-2
Phosphoserine aminotransferase	SERC_HUMAN	-2.5
PRA1 family protein 3	PRAF3_HUMAN	-2
Prefoldin subunit 3	PFD3_HUMAN	-20
Pre-mRNA-processing-splicing factor 8	PRP8_HUMAN	-5
Prenylcysteine oxidase 1	PCYOX_HUMAN	-5
Probable ATP-dependent RNA helicase	DDX17_HUMAN	-2.5
Probable serine carboxypeptidase	CPVL_HUMAN	-14.2
Programmed cell death 6-interacting protein	PDC6I_HUMAN	2.2
Programmed cell death protein 5	PDCD5_HUMAN	-25
Prohibitin-2	PHB2_HUMAN	-2.5
Prolactin regulatory element-binding protein	PREB_HUMAN	-14.2
Prolyl endopeptidase	PPCE_HUMAN	4.3
ProSAAS	PCSK1_HUMAN	2.6
Proteasome subunit alpha type-3	PSA3_HUMAN	-2.5
Proteasome subunit alpha type-4	PSA4_HUMAN	-5
Proteasome subunit beta type	PSB5_HUMAN	-3.3
Proteasome subunit beta type-6	PSB6_HUMAN	-33
Protein AMBP	AMBP_HUMAN	3.2
Protein CDV3 homolog	CDV3_HUMAN	-2
Protein CutA	CUTA_HUMAN	-2
Protein FAM98B	FA98B_HUMAN	-2.5
Protein NDRG1	NDRG1_HUMAN	-10
Protein NipSnap homolog 2	NIPS2_HUMAN	7.1
Protein phosphatase 1 regulatory subunit	PP1R7_HUMAN	-2.5
Protein sel-1 homolog 1	SE1L1_HUMAN	-5
Protein transport protein	SC16A_HUMAN	-3.3
Protein transport protein Sec24	SC24C_HUMAN	-10
Protein-glutamine gamma-glutamyltransferase	TGM2_HUMAN	2.1
Protein-L-isoaspartate(D-aspartate) O-methyltransferasePCMT1	PIMT_HUMAN	-2.5
Protein-tyrosine sulfotransferase 2	TPST2_HUMAN	-2.5
Prothymosin alpha	PTMA_HUMAN	2.9
Putative adenosylhomocysteinase 2	SAHH2_HUMAN	-10
Putative pre-mRNA-splicing factor ATP-dependent RNA helicase	DHX15_HUMAN	-3.3
Putative RNA-binding protein	RBM3_HUMAN	-2.5

Supplementary Table 6 continued.

Putative tropomyosin alpha-3 chain-like protein	TPM3L_HUMAN	2.4
Pyridoxine-5'-phosphate oxidase	PNPO_HUMAN	-10
Pyruvate dehydrogenase E1 component subunit beta	ODPB_HUMAN	-2
Ras suppressor protein 1	RSU1_HUMAN	4.9
Regenerating islet-derived protein 3-alpha	REG3A_HUMAN	-2.5
Regucalcin	RGN_HUMAN	-10
Reticulocalbin-1	RCN1_HUMAN	-20
Rho GDP-dissociation inhibitor 2	GDIR2_HUMAN	-10
Rho GTPase-activating protein 1	RHG01_HUMAN	-3.3
Ribonuclease	UK114_HUMAN	-5
Ribose-phosphate pyrophosphokinase 1	PRPS1_HUMAN	-10
RNA-binding protein	FUS_HUMAN	2.7
RNA-binding protein 8	RBM8A_HUMAN	-16.6
RNA-binding protein E	EWS_HUMAN	-3.3
Selenocysteine lyase	SCLY_HUMAN	7.2
Septin-2	SEPT2_HUMAN	-2
Serine hydroxymethyltransferase	GLYM_HUMAN	-5
Serine/arginine-rich splicing factor 2	SRSF2_HUMAN	-25
Serine/threonine-protein phosphatase 2A	PP2AA_HUMAN	-2.5
Serpin H1	SERPH_HUMAN	-5
Serpin I2	SPI2_HUMAN	-2
Serum amyloid P	SAMP_HUMAN	2.8
S-formylglutathione hydrolase	ESTD_HUMAN	-2.5
SH3 domain-binding glutamic acid-rich-like protein	SH3L1_HUMAN	-3.3
Short/branched chain specific acyl-CoA dehydrogenase	ACDSB_HUMAN	-2
Signal recognition particle 9	SRP09_HUMAN	2.6
Small nuclear ribonucleoprotein	SMD2_HUMAN	-10
Spermidine synthase	SPEE_HUMAN	-2.5
Spliceosome RNA helicase	DX39B_HUMAN	-10
Splicing factor 1	SF01_HUMAN	-10
Splicing factor U2AF	U2AF2_HUMAN	2.6
Splicing factor U2AF	U2AF1_HUMAN	-16.6
Sulfide:quinone oxidoreductase	SQRD_HUMAN	-3.3
Surfeit locus protein 4	SURF4_HUMAN	-3.3
T-complex protein 1 subunit alpha	TCPA_HUMAN	-2
Tetratricopeptide repeat protein 38	TTC38_HUMAN	-2
Thioredoxin domain-containing protein 17	TXD17_HUMAN	4.8
Thiosulfate sulfurtransferase/rhodanese-like domain-protein	TSTD1_HUMAN	-5
Trans-2,3-enoyl-CoA reductase	TECR_HUMAN	-2.5
Transcription factor BTF3 homolog 4	BT3L4_HUMAN	-33
Transforming growth factor-beta-induced protein	BGH3_HUMAN	-5
Translocon-associated protein subunit beta	SSRB_HUMAN	-5

Supplementary Table 6 continued.

Transmembrane 9 superfamily member 2	TM9S2_HUMAN	-3.3
Transmembrane emp24 domain-containing protein 7	TMED7_HUMAN	-3.3
Transmembrane protein 205	TM205_HUMAN	-10
Trifunctional purine biosynthetic protein adenosine	PUR2_HUMAN	-3.3
tRNA-splicing ligase	RTCB_HUMAN	-2.5
Tropomyosin alpha-1	TPM1_HUMAN	2.2
Tubulin beta-6	TBB6_HUMAN	-2.5
Tumor-associated calcium signal transducer 2	TACD2_HUMAN	-20
U2 small nuclear ribonucleoprotein A	RU2A_HUMAN	7.1
Ubiquitin carboxyl-terminal hydrolase 14	UBP14_HUMAN	-5
Ubiquitin-like modifier-activating enzyme 5	UBA5_HUMAN	-3.3
UPF0577 protein	K1324_HUMAN	-11
Vacuolar protein sorting-associated protein 35	VPS35_HUMAN	-2
V-type proton ATPase catalytic subunit A	VATA_HUMAN	-10
Xaa-Pro aminopeptidase	XPP1_HUMAN	-2
X-ray repair cross-complementing protein 6	XRCC6_HUMAN	-2
Zyxin	ZYX_HUMAN	-3.3
