# Supplementary material for: Proteomic Analysis of Disease Stratified Human Pancreas Tissue Indicates Unique Signature of Type 1 Diabetes
Source: PLoS One. 2015 Aug 24;10(8):e0135663. doi: 10.1371/journal.pone.0135663 (PMC4547762; doi:10.1371/journal.pone.0135663)
Supplement: S7 Table — (PDF) [file pone.0135663.s017.pdf]

S7 Table. List of genes represented in the network for differentially expressed proteins in AAb+ versus ND in figure 4A.

Symbol	Gene Name
26s Proteasome	26S proteosome
API5	apoptosis inhibitor 5
APOH	apolipoprotein H (beta-2-glycoprotein I)
ARF6	ADP-ribosylation factor 6
C3	complement component 3
C9	complement component 9
CD59	CD59 molecule, complement regulatory protein
CORO1B	coronin, actin binding protein, 1B
CTSB	cathepsin B
ELANE	elastase, neutrophil expressed
ERK	p42/44 mapk
ERK1/2	p42/p44 Map kinase
IFN Beta	Interferon beta
ILK	integrin-linked kinase
Jnk	JUN KINASE
LBP	lipopolysaccharide binding protein
LCN2	lipocalin 2
LTF	lactotransferrin
MAVS	mitochondrial antiviral signaling protein
MIF	macrophage migration inhibitory factor
MMP9	matrix metallopeptidase 9
MPO	myeloperoxidase
MYLK	myosin light chain kinase
NAMPT	nicotinamide phosphoribosyltransferase
NDRG1	N-myc downstream regulated 1
NFkB (complex)	transcription factor nuclear factor κ b
P38 MAPK	p38 MAP KINASE
Pkc(s)	Protein Kinase C
POSTN	periostin, osteoblast specific factor
PSMB5	proteasome (prosome, macropain) subunit, beta type, 5
PSMB6	proteasome (prosome, macropain) subunit, beta type, 6
PSMC5	proteasome (prosome, macropain) 26S subunit, ATPase, 5
SLC9A3R1	solute carrier family 9, subfamily A
TRIM25	tripartite motif containing 25
TXN	thioredoxin
