# Supplementary material for: Proteomic Analysis of Disease Stratified Human Pancreas Tissue Indicates Unique Signature of Type 1 Diabetes
Source: PLoS One. 2015 Aug 24;10(8):e0135663. doi: 10.1371/journal.pone.0135663 (PMC4547762; doi:10.1371/journal.pone.0135663)
Supplement: S8 Table — (PDF) [file pone.0135663.s018.pdf]

S8 Table. List of genes represented in the network for differentially expressed proteins in T1D versus ND in figure 5A.

Symbol	Gene Name
ANKRD1	ankyrin repeat domain 1 (cardiac muscle)
ANPEP	alanyl (membrane) aminopeptidase
APEX1	APEX nuclease (multifunctional DNA repair enzyme) 1
ATF4	activating transcription factor 4
BGN	biglycan
BHLHE40	basic helix-loop-helix family, member e40
CCL5	chemokine (C-C motif) ligand 5
CIITA	class II, major histocompatibility complex, transactivator
CXCL8	chemokine (C-X-C motif) ligand 8
EHMT2	euchromatic histone-lysine N-methyltransferase 2
Fc gamma receptor	Fcr gamma chain
GFRA1	GDNF family receptor alpha 1
HLA-C	major histocompatibility complex, class I, C
HLA-DRA	major histocompatibility complex, class II, DR alpha
Hla-Drb	Hla-Dr Beta
IFNG	interferon, gamma
IKBKE	inhibitor of kappa light polypeptide gene enhancer in B-cells, kinase epsilon
ITGB3	integrin, beta 3 (platelet glycoprotein IIIa, antigen CD61)
KIR	Killer Cell Immunoglobulin like receptor
MECP2	methyl CpG binding protein 2
NFKB1	nuclear factor of kappa light polypeptide gene enhancer in B-cells 1
PML	promyelocytic leukemia
POU2F1	POU class 2 homeobox 1
PTGS2	prostaglandin-endoperoxide synthase 2 (prostaglandin G/H synthase and cyclooxygenase)
PTMA	prothymosin, alpha
RFX5	regulatory factor X, 5 (influences HLA class II expression)
Rock	Rho Kinase
SMAD7	SMAD family member 7
SPHK1	sphingosine kinase 1
SYK	spleen tyrosine kinase
TGFB1	transforming growth factor, beta 1
TXN	thioredoxin
TYMP	thymidine phosphorylase
VASP	vasodilator-stimulated phosphoprotein
VTN	vitronectin
