# Supplementary material for: Proteomic Analysis of Disease Stratified Human Pancreas Tissue Indicates Unique Signature of Type 1 Diabetes
Source: PLoS One. 2015 Aug 24;10(8):e0135663. doi: 10.1371/journal.pone.0135663 (PMC4547762; doi:10.1371/journal.pone.0135663)
Supplement: S9 Table — (PDF) [file pone.0135663.s019.pdf]

S9 Table. List of genes represented in the network for differentially expressed proteins in T2D versus ND in Supplemental Figure 6.

Symbol	Gene Name
ACTA2	actin, alpha 2
ACTN1	actinin, alpha 1
ADAM9	ADAM metallopeptidase domain 9
AKR1C3	aldo-keto reductase family 1, member C3
BMP2	bone morphogenetic protein 2
BMP4	bone morphogenetic protein 4
CALR	calreticulin
CAT	catalase
CNN1	calponin 1, basic, smooth muscle
COL18A1	collagen, type XVIII, alpha 1
ENG	endoglin
ERK1/2	p42/44 mapk
FHL2	four and a half LIM domains 2
FN1	fibronectin 1
GPIIB-IIIA	Fibrinogen Receptor
HLA-A	major histocompatibility complex, class I, A
IFN Beta	Interferon beta
ITGB1	integrin, beta 1
Lh	Luteinizing Hormone
MT-CO2	cytochrome c oxidase subunit II
NFkB (complex)	transcription factor nuclear factor κ b
NPC2	Niemann-Pick disease, type C2
PDCD5	programmed cell death 5
PDLIM1	PDZ and LIM domain 1
PPAP2B	phosphatidic acid phosphatase type 2B
PSMD3	proteasome (prosome, macropain) 26S subunit
PTGER2	prostaglandin E receptor 2 (subtype EP2)
PTGER4	prostaglandin E receptor 4 (subtype EP4)
SF1	splicing factor 1
SHARPIN	SHANK-associated RH domain interactor
STAT1	signal transducer and activator of transcription 1
TLR4	toll-like receptor 4
ZYX	zyxin
