# Supplementary material for: Proteomic Analysis of Disease Stratified Human Pancreas Tissue Indicates Unique Signature of Type 1 Diabetes
Source: PLoS One. 2015 Aug 24;10(8):e0135663. doi: 10.1371/journal.pone.0135663 (PMC4547762; doi:10.1371/journal.pone.0135663)
Supplement: S10 Table — (PDF) [file pone.0135663.s020.pdf]

S10 Table. List of genes represented in the network for uniquely upregulated proteins in AAb+ versus ND in S7 Fig.

Symbol	Gene Name
ATF2	activating transcription factor 2
AXL	AXL receptor tyrosine kinase
CALD1	caldesmon 1
CD36	CD36 molecule (thrombospondin receptor)
CD40LG	CD40 ligand
CREB3	cAMP responsive element binding protein 3
DDB2	damage-specific DNA binding protein 2
DEFB103B	defensin, beta 103B
ELANE	elastase, neutrophil expressed
FCER1A	Fc fragment of IgE
GSTM1	glutathione S-transferase mu 1
Hsp90	heat shock protein 90
Ige	immunoglobulin E
Ikb	I Kappa B
IL13RA2	interleukin 13 receptor, alpha 2
LBP	lipopolysaccharide binding protein
LCN2	lipocalin 2
miR-491-5p	Micro RNA-491
MMP9	matrix metallopeptidase 9
MYLK	myosin light chain kinase
NAMPT	nicotinamide phosphoribosyltransferase
NFkB (complex)	transcription factor nuclear factor κ b
NFKBIZ	nuclear factor of kappa light polypeptide gene enhancer in B-cells inhibitor
PCMT1	protein-L-isoaspartate (D-aspartate) O-methyltransferase
PPARG	peroxisome proliferator-activated receptor gamma
PROC	protein C
RNASE3	ribonuclease, RNase A family, 3
SDC4	syndecan 4
SENP1	SUMO1/sentrin specific peptidase 1
STAT1	signal transducer and activator of transcription 1
STX4	syntaxin 4
TFPI2	tissue factor pathway inhibitor 2
TNFRSF18	tumor necrosis factor receptor superfamily
