# Supplementary material for: Proteomic Analysis of Disease Stratified Human Pancreas Tissue Indicates Unique Signature of Type 1 Diabetes
Source: PLoS One. 2015 Aug 24;10(8):e0135663. doi: 10.1371/journal.pone.0135663 (PMC4547762; doi:10.1371/journal.pone.0135663)
Supplement: S11 Table — (PDF) [file pone.0135663.s021.pdf]

S11 Table. List of genes represented in the network for uniquely upregulated proteins in T1D versus ND in S8 Fig.

Symbol	Gene Name
ADD1	adducin 1 (alpha)
ALOX5	arachidonate 5-lipoxygenase
APEX1	APEX nuclease 1
BGN	biglycan
C5	complement component 5
C7	complement component 7
CCND1	cyclin D1
CXCL8	chemokine (C-X-C motif) ligand 8
ENPP1	ectonucleotide pyrophosphatase/phosphodiesterase 1
Fc gamma receptor	Fcr gamma chain
GPER1	G protein-coupled estrogen receptor 1
HLA-C	major histocompatibility complex, class I, C
HLA-DRA	major histocompatibility complex, class II, DR alpha
HPRT1	hypoxanthine phosphoribosyltransferase 1
IKBKE	inhibitor of kappa light polypeptide gene enhancer in B-cells
IL1B	interleukin 1, beta
KIR	Killer Cell Immunoglobulin like receptor
LILRB1	leukocyte immunoglobulin-like receptor, subfamily B
LY6E	lymphocyte antigen 6 complex, locus E
MAPK1	mitogen-activated protein kinase 1
MECP2	methyl CpG binding protein 2
NFIX	nuclear factor I/X (CCAAT-binding transcription factor)
NOP58	NOP58 ribonucleoprotein
PIKFYVE	phosphoinositide kinase
PML	promyelocytic leukemia
POU2F1	POU class 2 homeobox 1
PRDX6	peroxiredoxin 6
Rnr	Ribosomal DNA Repeat Unit
RPS26	ribosomal protein S26
RPS6KA1	ribosomal protein S6 kinase
SPHK1	sphingosine kinase 1
TGFB1	transforming growth factor, beta 1
TYMP	thymidine phosphorylase
