# Supplementary material for: Proteomic Analysis of Disease Stratified Human Pancreas Tissue Indicates Unique Signature of Type 1 Diabetes
Source: PLoS One. 2015 Aug 24;10(8):e0135663. doi: 10.1371/journal.pone.0135663 (PMC4547762; doi:10.1371/journal.pone.0135663)
Supplement: S12 Table — (PDF) [file pone.0135663.s022.pdf]

S12 Table. List of genes represented in the network for uniquely upregulated proteins in T2D versus ND in S9 Fig.

Symbol	Gene Name
ACAT1	acetyl-CoA acetyltransferase 1
AKR1C3	aldo-keto reductase family 1, member C3
CAPN2	calpain 2
CD300C	CD300c molecule
CNBP	CCHC-type zinc finger
CPE	carboxypeptidase E
CSRNP1	cysteine-serine-rich nuclear protein 1
DAG1	dystroglycan 1
DAPK2	death-associated protein kinase 2
DLEU1	deleted in lymphocytic leukemia 1
EHD1	EH-domain containing 1
ESR1	estrogen receptor 1
FABP4	fatty acid binding protein 4
GSTM1	glutathione S-transferase mu 1
GSTM2	glutathione S-transferase mu 2
HSD17B11	hydroxysteroid (17-beta) dehydrogenase 11
LILRA5	leukocyte immunoglobulin-like receptor, subfamily A (with TM domain)
mir-8	microRNA 200a
MMP8	matrix metallopeptidase 8 (neutrophil collagenase)
MT-CO2	cytochrome c oxidase subunit II
NLRC4	NLR family, CARD domain containing 4
NR3C1	nuclear receptor subfamily 3, group C, member 1
PDCD6IP	programmed cell death 6 interacting protein
PGR	progesterone receptor
PNRC1	proline-rich nuclear receptor coactivator 1
PPARG	peroxisome proliferator-activated receptor gamma
PTGS1	prostaglandin-endoperoxide synthase 1 (prostaglandin G/H synthase and cyclooxygenase)
SDC4	syndecan 4
SLC7A2	solute carrier family 7 (cationic amino acid transporter, y+ system), member 2
SRP9	signal recognition particle 9kDa
SUZ12	SUZ12 polycomb repressive complex 2 subunit
TGM2	transglutaminase 2
TNF	tumor necrosis factor
